# Integrated Analysis of Microarray and RNA-Seq Data for the Identification of Hub Genes and Networks Involved in the Pancreatic Cancer

**DOI:** 10.3389/fgene.2021.663787

**Published:** 2021-06-23

**Authors:** Maryum Nisar, Rehan Zafar Paracha, Iqra Arshad, Sidra Adil, Sabaoon Zeb, Rumeza Hanif, Mehak Rafiq, Zamir Hussain

**Affiliations:** ^1^Research Centre for Modeling and Simulation (RCMS), National University of Sciences and Technology (NUST), Islamabad, Pakistan; ^2^Atta-ur-Rahman School of Applied Biosciences-ASAB, National University of Sciences and Technology (NUST), Islamabad, Pakistan

**Keywords:** pancreatic cancer, co-expression network, biomarker, therapeutic target, differential expression, TCGA, enrichment analysis, focal adhesion pathway

## Abstract

Pancreatic cancer (PaCa) is the seventh most fatal malignancy, with more than 90% mortality rate within the first year of diagnosis. Its treatment can be improved the identification of specific therapeutic targets and their relevant pathways. Therefore, the objective of this study is to identify cancer specific biomarkers, therapeutic targets, and their associated pathways involved in the PaCa progression. RNA-seq and microarray datasets were obtained from public repositories such as the European Bioinformatics Institute (EBI) and Gene Expression Omnibus (GEO) databases. Differential gene expression (DE) analysis of data was performed to identify significant differentially expressed genes (DEGs) in PaCa cells in comparison to the normal cells. Gene co-expression network analysis was performed to identify the modules co-expressed genes, which are strongly associated with PaCa and as well as the identification of hub genes in the modules. The key underlaying pathways were obtained from the enrichment analysis of hub genes and studied in the context of PaCa progression. The significant pathways, hub genes, and their expression profile were validated against The Cancer Genome Atlas (TCGA) data, and key biomarkers and therapeutic targets with hub genes were determined. Important hub genes identified included ITGA1, ITGA2, ITGB1, ITGB3, MET, LAMB1, VEGFA, PTK2, and TGFβ1. Enrichment analysis characterizes the involvement of hub genes in multiple pathways. Important ones that are determined are ECM–receptor interaction and focal adhesion pathways. The interaction of overexpressed surface proteins of these pathways with extracellular molecules initiates multiple signaling cascades including stress fiber and lamellipodia formation, PI3K-Akt, MAPK, JAK/STAT, and Wnt signaling pathways. Identified biomarkers may have a strong influence on the PaCa early stage development and progression. Further, analysis of these pathways and hub genes can help in the identification of putative therapeutic targets and development of effective therapies for PaCa.

## 1. Introduction

Pancreatic cancer (PaCa) is the 11th most common and the seventh most fatal malignancy worldwide with 459,000 new cases and 432,000 deaths in the year 2018 (Bray et al., 2018). This is because usually patients exhibit symptoms in advanced stages. Symptoms of PaCa are nonspecific including jaundice, abdominal pain, nausea, weight loss, dark colored urine, pale stool, and depression, making its diagnosis difficult (Rawla et al., 2019). In majority of the patients, PaCa leads to the metastasis and pancreatic exocrine insufficiency (PEI), which eventually results in the development of metabolic abnormalities (Li et al., 2004).

Pancreatic neuroendocrine tumor and Pancreatic adenocarcinoma are two types of PaCa. The pancreatic neuroendocrine tumor develops in the cells of endocrine gland (islets of Langerhans) responsible for releasing multiple hormones such as insulin, glucagon, somatostatin, and polypeptide. Pancreatic adenocarcinoma also known as pancreatic ductal adenocarcinoma (PDAC) develops in the ductal tissues of the pancreas exocrine gland (De La Cruz et al., 2014; Hidalgo et al., 2015). It is the most prevalent type of PaCa with more than 95% cases, very poor early diagnosis, and high mortality rate. About 95% of the patients died in the first year of diagnosis, and only 5% of patients survive up to 5 years (Hidalgo et al., 2015). The developmental phases of cancer are classified in various clinical stages. The stages 0, IA, and IB are localized, non-invasive, and resectable lesions, whereas stages IIA and IIB are localized, invasive, and resectable tumor. Stage III is localized, advanced, and unresectable phase of PaCa, while stage IV is metastatic phase (De La Cruz et al., 2014).

Risk factors of PaCa include demographic (age, sex, and region), hereditary, and environmental factors. PaCa is commonly diagnosed at the age of 50. Males are more susceptible to develop PaCa than females. The European population is more prone to establish PaCa than other populations (Li et al., 2004; Bray et al., 2018). Smoking, alcohol consumption, and sedentary lifestyle increase the chances of its development (De La Cruz et al., 2014; Rawla et al., 2019). Prolonged chronic pancreatitis is also a major risk factor for the development and progression of PaCa (Rawla et al., 2019).

PaCa cells exhibit mutations and deregulation of different genes resulting in metastasis and causes chemotherapy resistance. Mutations in codon number 12 or increased expression of oncogene KRAS are common in PaCa patients (Smit et al., 1988; Yu et al., 2010). Overexpressed KRAS triggers the hedgehog signaling pathway, along with activation of MAP2K4 and RASGRP3 (Ji et al., 2007). In advanced stages of disease, underexpression of SMAD4, INK4a/ARF, and TP53 genes is also well-reported (Zhang et al., 2019). Overexpression of various other genes are also reported in PaCa such as MYB, which is a key factor for tumor progression and metastases (Srivastava et al., 2015). The SOX9 (Grimont et al., 2015) and HIF-1α cause hypoxia and reduce anti-cancer drug delivery in the tumor region (Spivak-Kroizman et al., 2013). Other core signaling pathways activated in PaCa includes apoptosis, Wnt/Notch, transforming growth factor-β (TGF-β) (Hidalgo et al., 2015), and phosphatidylinositol 3-kinase PI3K/Akt pathway (Hill et al., 2010). GPR87 overexpressed in PaCa activates NF-κB signaling pathway, which alternatively enhances the cancer progression. Its overexpression is also reported in multiple other cancer types (Wang et al., 2017). Vascular endothelial growth factor receptor VEGFR-2 of VEGF-A family has been also identified in PaCa patients (Costache et al., 2015). GPR87, FN14, and VEGF genes are key contributors in cell proliferation, angiogenesis, migration, and initiate metastases (Han et al., 2005; Costache et al., 2015; Wang et al., 2017).

There are multiple therapies employed for the treatment of PaCa including resection, chemotherapy, adjuvant chemotherapy, targeted therapies, and target specific immunotherapies (Seicean et al., 2015). The adjuvant chemotherapy is the combination of resection, radiation, or targeted therapy with chemotherapy (Neoptolemos et al., 2001). FOLFIRINOX is Food and Drug Administration (FDA)-approved therapy for locally advanced and metastasized PaCa. It is the combination of drugs including leucovorin calcium (folinic acid), fluorouracil, irinotecan, and oxaliplatin. FOLFIRINOX is used prior to resection for reducing the size of the tumor in the patients with locally advanced stages. Its overall response rates (ORRs) are <28% with 11 months without cancer progression (Faris et al., 2013). In targeted therapy, various kinases, cancer specific proteins, and receptors are targeted. Passive immunotherapy is also a type of targeted therapy, in which monoclonal antibodies are infused in the patients. Drugs against EGFR, HER2, VEGF, MAPK, IGF-1R, c-Met, and PI3K/Akt/mTOR are under consideration in different clinical trials (Borja-Cacho et al., 2008).

Despite of all these therapies, patients have a very low survival rate. This happens because of the late diagnosis of PaCa due to non-specific symptoms and very low efficacy of drugs (Seicean et al., 2015). So, the major concern is early diagnosis, which can be done by identifying PaCa-specific biomarkers and effective prognosis techniques. There is a strong need for the development of effective anti-PaCa drug, with low side effect and high cancer specific targeting. In this study, extensive transcriptome profiling of PaCa has been performed. Microarray and high-throughput sequencing data are significantly contributing in understanding the molecular changes occurring in cells during disease development and progression.The main focus of this study is a holistic gene expression profiling and co-expression analysis of genes in PaCa. Another objective of this study is to identify therapeutic targets for targeted cancer therapy out of these disease-related significant genes.

Important pathways identified for the significant genes determined by DE analysis are ECM–receptor interaction, HIF-1 signaling pathway, pathways in cancer, focal adhesion (FA), PI3K-Akt signaling pathway, and amoebiasis. Along with DE analysis, genes co-expression network analysis of data was performed to identify patterns or modules of genes associated with cancer phenotype. Hub genes identified out of modules show significant association with PaCa. These hub genes show involved in PaCa, progression pathways ECM–receptor interaction, and FA, and also involved as key entities in pathways identified through DE analysis. To further validate these findings, we performed The Cancer Genome Atlas (TCGA) RNA-seq data analysis, and TCGA results exhibit strong concordance with results of above two methodologies. Additionally, the expression of identified biomarkers in normal pancreas tissue is studied to validate their causality in PaCa.

## 2. Methodology

**Figure 1:**
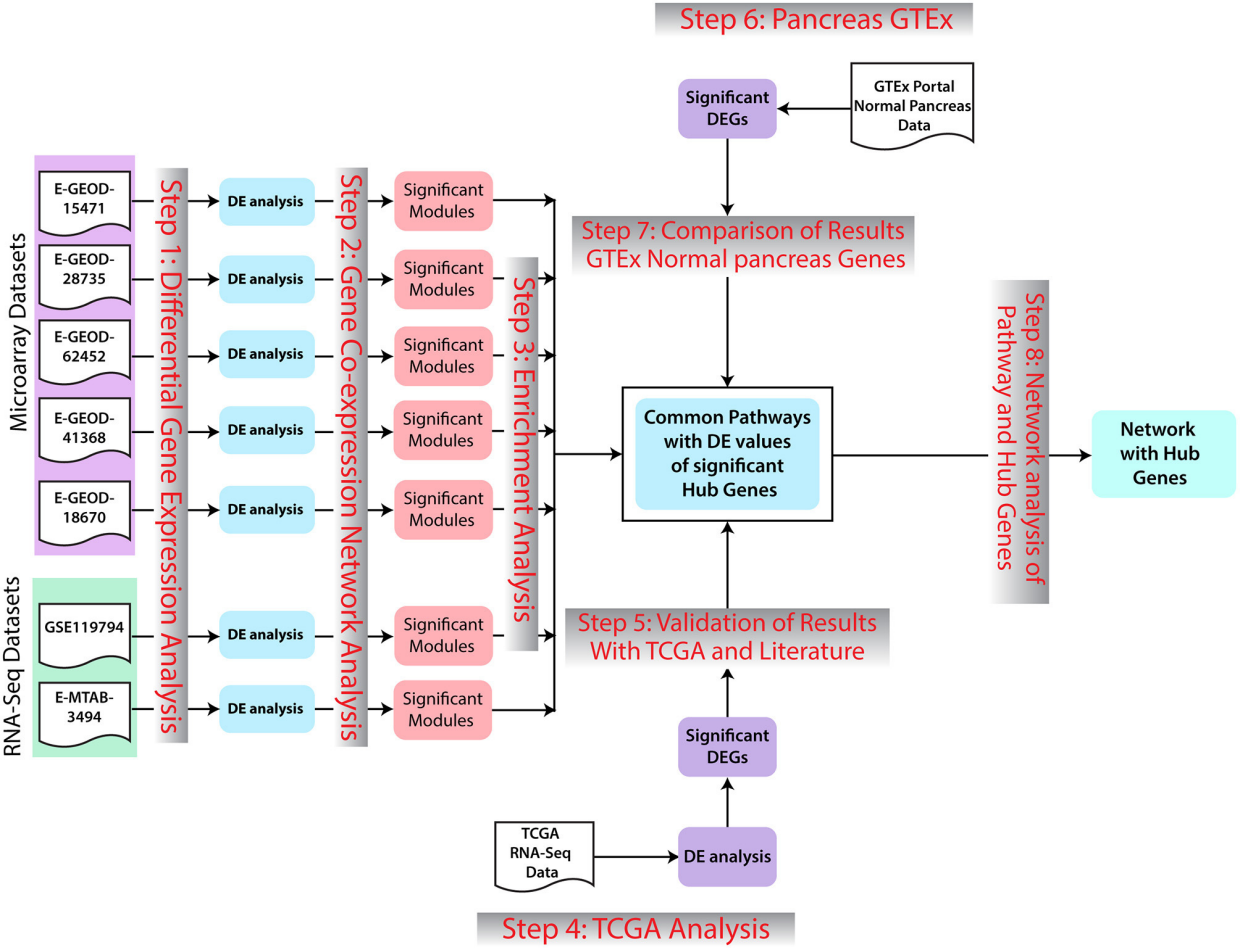
Methodology workflow. In the first step, differential expression analysis was performed to identify significant differentially expressed genes (DEGs). Afterward, for further analysis of genomic data, co-expression network analysis of DEGs with −0.5>*log*_2_*FC*>0.5 was preformed. Significant modules for each dataset were selected. Enrichment analysis of genes of selected modules was preformed. Additionally, the common pathways in all datasets were further studied regarding pancreatic cancer (PaCa) development. Results were validated with The Cancer Genome Atlas (TCGA) cohort analysis, GTEx Portal, and literature. In the last step, gene network interaction analysis and hub genes identification were performed.

The overall workflow of this study includes identification of DEGs in PaCa using multiple microarray and RNA-seq datasets. Important pathways are then determined on the basis of enrichment analysis of these DEGs. Moreover, WGCNA analysis was performed to determine hub genes, which could be putative therapeutic targets. TCGA analysis was performed to validate the results. The overall workflow of the study is given in Figure 1.

### 2.1. Datasets Inclusion Criteria

The microarray and RNA-seq datasets were collected from EMBL-EBI (https://www.ebi.ac.uk/) and GEO (https://www.ncbi.nlm.nih.gov/geo/) against query words such as PaCa and pancreatic ductal adenocarcinoma (PDAC), visited on March 2020. Selection criteria were based on the fact that datasets must be from the origin of *Homo Sapiens*. Moreover, the datasets consist of samples collected from patient tissue region of PaCa tumor and adjacent healthy pancreatic region, excluding cell lines-based experimentation. Besides this, selected datasets were free from any therapy or drug, mutations, induced gene expression, or gene knockdown. All datasets were selected with enough number of samples of PaCa and control to obtain statistically significant results.

For microArray analysis, five datasets were selected on the basis of above selection criteria. Details of all datasets including accession number, samples information, and platform are provided in Table 1. The Dataset E-GEOD-18670 comprised of 24 samples including six circulating tumor cells (metastases), six haematological, six PDAC tumor tissues, and six adjacent normal tissue. Out of these samples, six PDAC tumor and 6 adjacent normal samples were analyzed in this study according to our sample inclusion criteria. The main objective of our study is to identify biomarkers or targets present on the surface of tumor cells. For RNA-seq analysis, two datasets were selected according to the above-mentioned selection criteria. All details of datasets are provided in Table 2. The dataset E-MTAB-3494 consists of 11 samples of total RNA-sequencing data, including five normal and six PDAC samples. The dataset GSE119794 comprises 20 mRNA samples, including 10 PDAC, 10 normal, and 20 miRNA (10 PDAC and 10 normal) samples. In this study, only mRNA samples were used for analysis.

**Table 1:** Microarray datasets.

**Sr.No**	**Accession number**	**Total samples**	**Selected samples**	**Platform**	**Country**	**References**
1	E-GEOD-15471	78 samples Sample types: •39 Normal •39 Tumor	78 samples Sample types: •39 Normal •39 Tumor	Affymetrix GeneChip Human Genome U133 Plus 2.0 [*HG*−*U*133_*Plus*_2]	Romania	Badea et al. (2008)
2	E-GEOD-28735	90 samples Sample Type: •45 Normal •45 Tumor	90 samples Sample Type: •45 Normal •45 Tumor	Affymetrix GeneChip Human Gene 1.0 ST Array [*HuGene*−1_0−*st*−*v*1]	USA	Zhang et al. (2012)
3	E-GEOD-62452	130 samples Sample type: •61 Normal •69 Tumor	130 samples Sample type: •61 Normal •69 Tumor	Affymetrix GeneChip Human Gene 1.0 ST Array [*HuGene*−1_0−*st*−*v*1]	USA	Yang et al. (2016)
4	E-GEOD-41368	12 samples Sample Type: •6 Normal •6 Tumor	12 samples Sample Type: •6 Normal •6 Tumor	Affymetrix GeneChip Human Gene 1.0 ST Array [*HuGene*−1_0−*st*−*v*1]	Italy	Frampton et al. (2014)
5	E-GEOD-18670	24 samples Sample Type: •6 Metastasis •6 Haematological •6 Normal •6 Tumor	12 samples Sample Type: •6 Normal •6 Tumor	Affymetrix GeneChip Human Genome U133 Plus 2.0 [*HG*−*U*133_*Plus*_2]	Belgium	Sergeant et al. (2012)

**Table 2:** RNA-seq datasets.

**Sr. No**	**Accession number**	**Total samples**	**Selected samples**	**Platform**	**Country**	**References**
1	E-MTAB-3494	11 samples Sample type: •5 Normal •6 Tumor	11 samples Sample type: •5 Normal •6 Tumor	Illumina HiSeq 2000 (Homo sapiens)	Germany	Müller et al. (2015)
2	GSE119794	40 samples Sample type: •10 Normal (mRNA) •10 Tumor (mRNA) •10 Normal (miRNA) •10 Tumor (miRNA)	20 samples Sample type: •10 Normal (mRNA) •10 Tumor (mRNA)	Illumina HiSeq 2000 (Homo sapiens)	China	Lin et al. (2019)

### 2.2. Differential Gene Expression Analysis

#### 2.2.1. Microarray Data Analysis

Quality control of data was performed using multiple data visualization and filtering techniques like Box plot, Relative Log Expression (RLE) plot, principal component analysis (PCA) plot, and heatmap. RLE plots were generated using the Bioconductor R package to visualize unwanted variation from gene median in high-dimensional microarray data (Gandolfo and Speed, 2018). The PCA plot and Heatmap were generated using Bioconductor R packages (Gu et al., 2016; Klaus and Reisenauer, 2018). These plots were used to analyze the clusters of samples with respect to the phenotypes. Then probes with low-intensity values were filtered to avoid noise in the data due to non-specific hybridization of probes and also duplicates were removed. Quantile normalization method was used for gene expression intensity normalization (Hicks and Irizarry, 2014; Hicks et al., 2017).

For the statistical analysis, linear regression model along with eBayes were used. The *limma* R package was used to fit a linear model for obtaining DEGs in PaCa samples relative to normal (Ritchie et al., 2015). Then “Empirical Bayes” model was fitted using an *eBayes* function in R to compute t-statistics that generates the statistical significance value (*p*-value) for each DE gene (Klaus and Reisenauer, 2018). Significant DEGs were obtained by applying p-value threshold of 0.05 and *Log*_2_*FC* (*Log*_2_ fold change) threshold (*Log*_2_*FC* < −1 and *Log*_2_*FC* > 1). To visualize DEGs, enhanced volcano plots of DEGs with *Log*_2_*FC* on x-axis and −*Log*_10_ (*p*-value) on y-axis were plotted (Blighe and Lewis, 2020).

#### 2.2.2. RNA-Seq Data Analysis

Quality assessment and preprocessing of sequencing data is a crucial step to perform high-level analysis. *Fastp* tool was utilized for data quality assessment, base correction, and removal of duplicates from the raw data of RNA-seq. It integrates the functionality of multiple tools including FASTQC, Cutadapt, Trimmomatic, and AfterQC, and faster than these stand alone tools (Chen et al., 2018). The next step was mapping and alignment of reads with reference genome. *HISAT2* (hierarchical indexing for spliced alignment of transcripts 2) tool was used to align reads with human reference genome (hg38). It rapidly searches genome using graph Ferragina–Manzini (FM) index and can align both DNA and RNA sequence reads. It can perform splice alignment of whole-genome, transcript, and exon much faster than TopHat2, Bowtie, and BWA (Liu et al., 2018). After the alignment, count data of expressed genes were generated using hg38 annotation gtf file in *htseq-count* tool (Anders et al., 2015). The read count data generated by *htseq-count* tool was then subjected to DEGs analysis using *DESeq* (Love et al., 2014). Reads with low expression values were filtered by discarding row entries with row sum <10. The *DESeq* uses median-of-ratios method for normalizing expression data. The filtering criteria used for obtaining set/list of statistically significant DEGs was *p*-value < 0.05, and −1> *Log*_2_*FC* > 1.

### 2.3. Co-expression Network Analysis

Co-expressed genes are considered to be involved in related signaling pathways and have similar biological function. Therefore, for the identification of co-expressed genes in PaCa, co-expression network analysis of microarray and RNA-seq datasets was performed using *WGCNA* Bioconductor R package (Langfelder and Horvath, 2008). For this analysis, list of DEGs with *p*-value < 0.05 and −0.5 > *Log*_2_*FC* > 0.5 was retrieved. Then quantile normalized expression values of these DEGs for microarray data and expression count table (RNA-seq platform) for RNA-seq data were used for further analysis. The *goodSamplesGenes* function was used for quality control of data by determining and removing deletions and outliers. The Soft threshold value (β) important for construction of scale-free network was determined by generating gene expression similarity matrix. The scale-free network means that data do not have any batch effects. The gene expression similarity matrix was generated by calculating Pearson correlation coefficient of each gene with all other genes in the data. Then adjacency and topology matrix, genetree (Denogram) with modules, and gene module membership along with gene phenotype correlation were calculated. Modules of significantly co-expressed and correlated genes were selected on the basis of high module trait correlation (0.6) and statistical significance (*p*-values < 0.05). Then genes with trait correlation >0.6 and gene module correlation >0.6 were selected for further analysis.

### 2.4. Gene List Enrichment Analysis

After the generation of gene list in above step, the next step was to compute gene enrichment for determination of functionally associated genes involved in different pathways and regulating the expression of other genes. For this purpose, first the lists of common underexpressed and overexpressed genes in all datasets were determined. Then both this DEGs list was annotated using Enrichr web server (https://maayanlab.cloud/Enrichr/). Enrichr is an open source web-based gene enrichment analysis tool; it integrates results from multiple libraries (Chen E. Y. et al., 2013). We utilized KEGG pathways option in Enrichr to identify a list of pathways against each gene list. Statistically significant pathways with Fisher exact test *p*-value < 0.05 and high combined score were selected. These important pathways were further explored with respect to therapeutic targets for PaCa.

### 2.5. Network Analysis of Identified Pathways and Hub Gene Determination

The string database STRING (https://string-db.org/) was used to retrieve the protein–protein interaction pattern and network (Szklarczyk et al., 2019). In STRING, the highest confidence score of (0.900) was used to generate interaction network. The interaction pattern generated was then downloaded and further analyzed in Cytoscape software (Otasek et al., 2019). In Cytoscape, Network Analyze module was used to evaluate the statistics and interaction profiles of all genes, which helps in hub gene determination. The MCODE app was utilized to identify dense interacting clusters out of whole network (Bader and Hogue, 2003). Then eight different topological analysis methods of CytoHubba were used to identify hub genes (biomarkers) (Chin et al., 2014; Ma et al., 2021). The used methods include Maximal Clique Centrality (MCC), Density of Maximum Neighborhood Component (DMNC), Maximum Neighborhood Component (MNC), Degree, Edge Percolated Component (EPC), Bottleneck, EcCentricity, and Closeness.

### 2.6. TCGA Analysis

To validate and improve reliability of our results, we analyzed PaCa gene expression data (RNA-seq) from TCGA database. The data searching, downloading, and preparation were performed using *TCGAbiolinks* Bioconductor R package (Colaprico et al., 2016). The total of 178 PaCa patients' data fulfilling the inclusion criteria of gene expression quantification and primary tumor samples were obtained from TCGA database. The level 1 raw count data were downloaded from the TCGA Data Portal belonging to the Illumina HiSeq sequencing platform. The quantile normalization was performed before analysis using *TCGAanalyze_Normalization* R function of *TCGAbiolinks* package. Then for the identification of differentially expressed genes in the PaCa patients, *edgeR* package from Bioconductor was utilized (Robinson et al., 2010). Genes were filtered using −1 > *Log*_2_*FC* > 1 and *P* < 0.05 values and were considered to indicate statistically significant differences.

### 2.7. GTEx Normal Pancreas Data Comparison

Along with the validation of cancer related genes from TCGA, expression of these genes was also compared with normal pancreas. The GTEx Protal (https://gtexportal.org/home/) was used to retrieve the gene expression profile of human pancreas tissues (GTEx Consortium, 2020).

## 3. Results

Differential expression analysis of microarray and RNA-seq datasets generate the lists of significant DEGs for every data, fulfilling the selection criteria of *Log*_2_*FC* and *p*-value. The number of total DEGs and overexpressed and underexpressed DEGs are given in Table 3. These DEGs for all datasets are graphically represented using the volcano plot in Supplementary Figures 1–7. The lists of DEGs along with their *Log*_2_*FC* values, average *Log*_2_*FC*s, and standard deviation are provided in Supplementary Table 1. General trend of differential expression result represents more overexpressed genes than the underexpressed. The list of common underexpressed genes in all datasets was generated and out of which seven genes were identified. The enrichment analysis of these genes was performed using Enrichr, which determined the involvement of these genes in 14 different pathways. The AOX1 is involved in the metabolism of Vitamin B6, nicotinate, nicotinamide, tyrosine, tryptophan, and retinol, along with valine, leucine, and isoleucine degradation. Underexpression of AOX1 gene represents the disruption of above important pathways in PaCa cells. The C5 and IAPP genes are predicted to be involved in neuroactive ligand-receptor interaction, *Staphylococcus aureus* infection, Pertussis, complement, and coagulation cascades. Cumulatively, 69 common overexpressed genes were detected. Enrichment analysis of these genes determined their activity in 26 significant pathways. About 13 genes such as MET, ITGA3, ITGA2, LAMA3, and SLC2A1 are predicted to be involved in multiple cancer-related pathways such as ECM–receptor interaction, small cell lung cancer, FA, central carbon metabolism in cancer, pathways in cancer, PI3K-Akt signaling pathway, renal cell carcinoma, and HIF-1 signaling pathway. Out of these overexpressed genes, multiple receptor proteins, transmembrane, transporter, and surface proteins were also predicted.

**Table 3:** Total number of DEGs retrieved from all microarray and RNA-seq datasets are presented against the number of over and underexpressed genes are shown against each dataset.

**Sr. No**	**Accession number**	**Total DEGs**	**Overexpressed** **DEGs**	**Underexpressed** **DEGs**
**Microarray Datasets**				
1	E-GEOD-15471	1,665	1,466	199
2	E-GEOD-28735	364	227	137
3	E-GEOD-62452	274	174	100
4	E-GEOD-41368	1,688	1,222	466
5	E-GEOD-18670	1,164	798	366
**RNA-seq Datasets**				
1	GSE119794	2,174	1,194	980
2	E-MTAB-3494	3,804	2,193	1611
**TCGA Data**				
1	TCGA-PAAD	1,514	674	840

**Figure 2:**
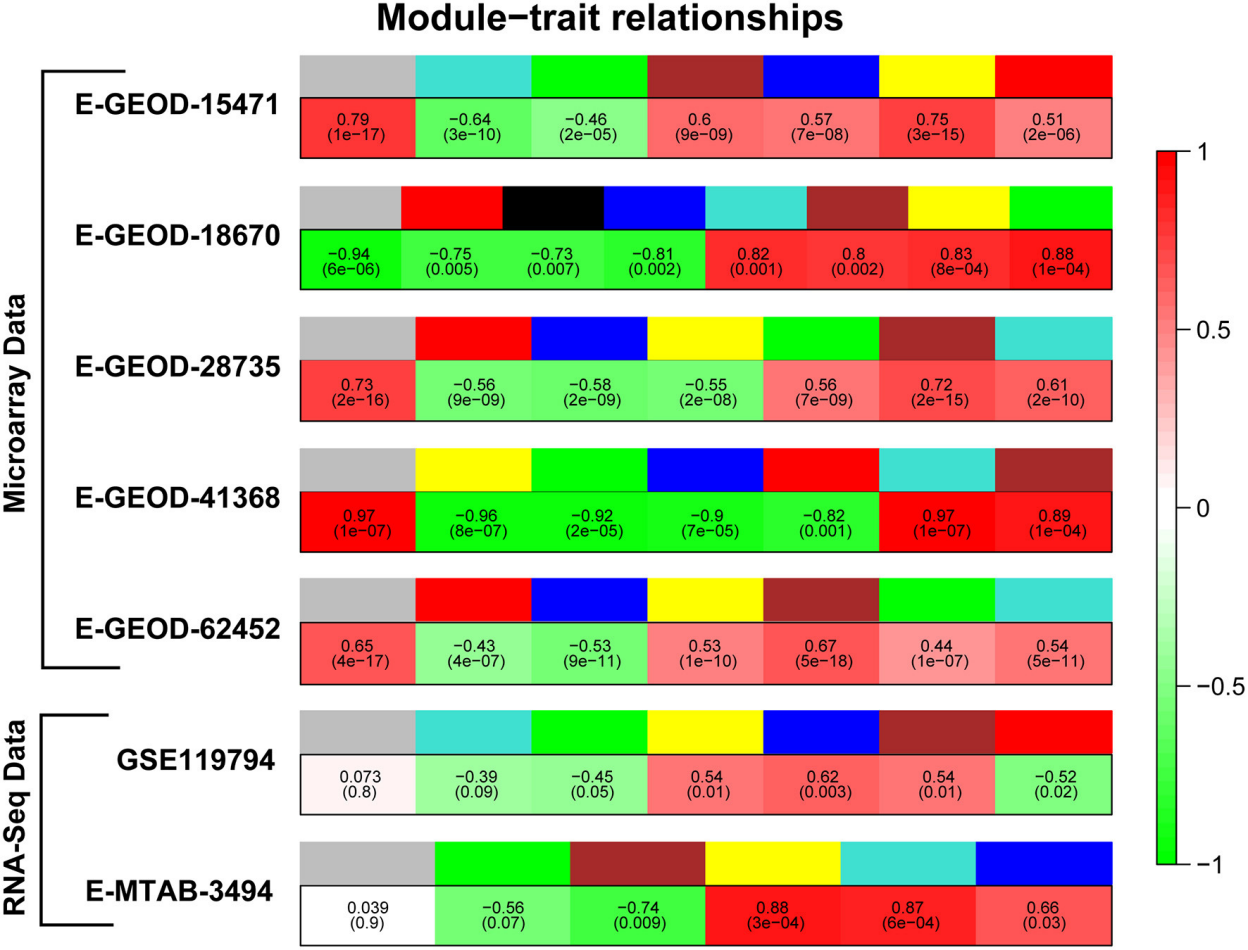
Correlation of modules with pancreatic cancer (PaCa). Figure represents module colors for all datasets and correlation of modules with PaCa phenotype. Colors of modules are given in upper row, whereas green and red colors in lower row represent negative and positive correlation, respectively.

The gene co-expression network analysis was performed to obtain clusters of co-expressed genes in PaCa cells. The reason of performing differential expression analysis along with gene co-expression network analysis was to obtain significant DEGs from all datasets and to also determine the co-expression of these DEGs. In this study, the main focus was to analyze the differential expression of receptors, which actually initiate cancer related pathways. The TCGA cohort analysis was also performed to validate the results. To enhance the reliability of co-expression network analysis results, 5 microarray and 2 RNA-seq datasets were analyzed. First, we extracted the list of differentially expressed genes with −0.5 ≤ *Log*_2_*FC* ≥ 0.5 values, so the number of genes for each dataset was different for this analysis. Then WGCNA analysis was performed separately for each dataset. First the soft threshold value for each dataset was determined. The soft threshold value or power value (β) was required for construction of scale free networks, in which few nodes/hub genes connected to more number of nodes than the peripheral ones. The β value from 2 to 20 is evaluated to obtain scale-free network on *R*^2^ ≥ 0.8. Plots for β value selection is provided for all datasets in Supplementary Figures 8–14, Part a. Then the adjacency matrix was generated using identified β value, afterward Topology Overlap Matrix (TOM) and gene cluster dendrogram along with their modules were generated. The module trait relationship was determined by calculating Pearson's correlation of module with PaCa. Figure 2 represents modules color and their respective correlation with PaCa for all datasets. Then modules with correlation ≥ 0.6 were selected, and according to set criteria three modules (gray, brown, and yellow) were selected for dataset E-GEOD-15471. Likewise, different number of modules were selected for remaining datasets, and names of selected modules are given in Table 4. The gene cluster dendrogram, trait-module relationship plots, and gene scatter plot of selected modules for all datasets are provided in Supplementary Figures 8–14, Part (b, c, d, etc.). Then the genes with correlation >0.6 with PaCa and module membership (MM) correlation >0.6 were filtered. Selected genes for all modules are provided in the first data sheet of Supplementary Table 2. Furthermore, modules for each dataset were merged into one. The enrichment of genes was performed using Enrichr, and the involvement of genes in cancer-related pathways was predicted. The significant pathways with *p*-value < 0.05 are given in Supplementary Table 3, and number of significant pathways are provided in Table 4. To further study the important hub genes involved in the significant pathways, common pathways in all datasets were selected.

**Table 4:** The table contains information related to WGCNA results, and second column contains name to datasets analyzed.

**Sr. No**	**Dataset name**	**β-value**	**Modules with** **correlation >= 0.6** **(Pos-Mod)**	**Number of** **significant** **genes** **(Pos-Mod)**	**Number of** **significant** **pathways** **(Pos-Mod)**	**Modules with** **Correlation** **< = −0.6** **(Neg-Mod)**	**Number of** **significant** **genes** **(Neg-Mod)**	**Number of** **significant** **pathways** **(Neg-Mod)**
**Microarray Data**								
1	E-GEOD-15471	20	Gray, brown, yellow	895	51	Turquoise	602	19
2	E-GEOD-18670	16	Turquoise, brown, yellow, Green	1,514	78	Gray, red, black, blue	659	42
3	E-GEOD-28735	9	Turquoise, gray, brown	166	33	0	Nill	Nill
4	E-GEOD-41368	16	Turquoise, gray, brown	2,457	151	Yellow, green, red, blue	741	40
5	E-GEOD-62452	10	Gray, brown	18	13	0	Nill	Nill
**RNA-seq Data**								
1	GSE119794	12	Blue	67	5	0	Nill	Nill
2	E-MTAB-3494	12	Yellow, turquoise, blue	1,985	133	Brown	343	28

*The third column consist of soft threshold value used for calculating adjacency matrix, and forth and seventh column show the names of modules choosed for further analysis. Remaining column contain information of genes selected and significant pathways retrieved for all datasets*.

**Figure 3:**
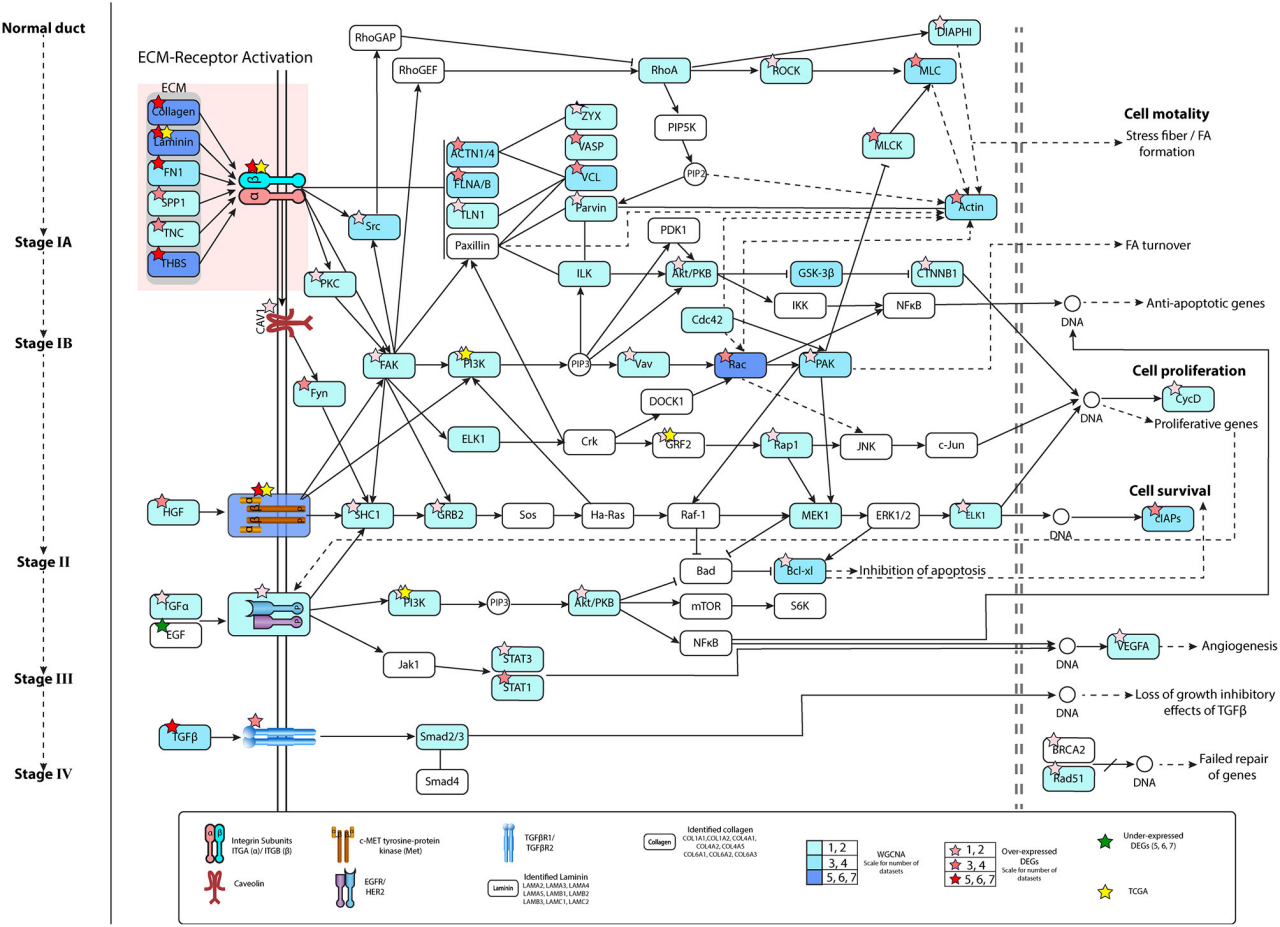
Pathway representing pancreatic cancer (PaCa) progression from normal pancreas cell to invasive stage of cancer. ECM molecules initiate multiple signaling pathways by interacting with integrin complexes. The growth factor receptors MET and EGFR/HER2 activation in initial stages of PaCa, activates signaling cascades, which lead to expression of anti-apoptotic, proliferative, and cell survival genes. Figure represent the information related to gene identified in number of datasets in WGCNA and differential gene expression (DE) analysis. Color filled in entities show the dataset number for WGCNA. Color of stars represent number of DE datasets.

**Figure 4:**
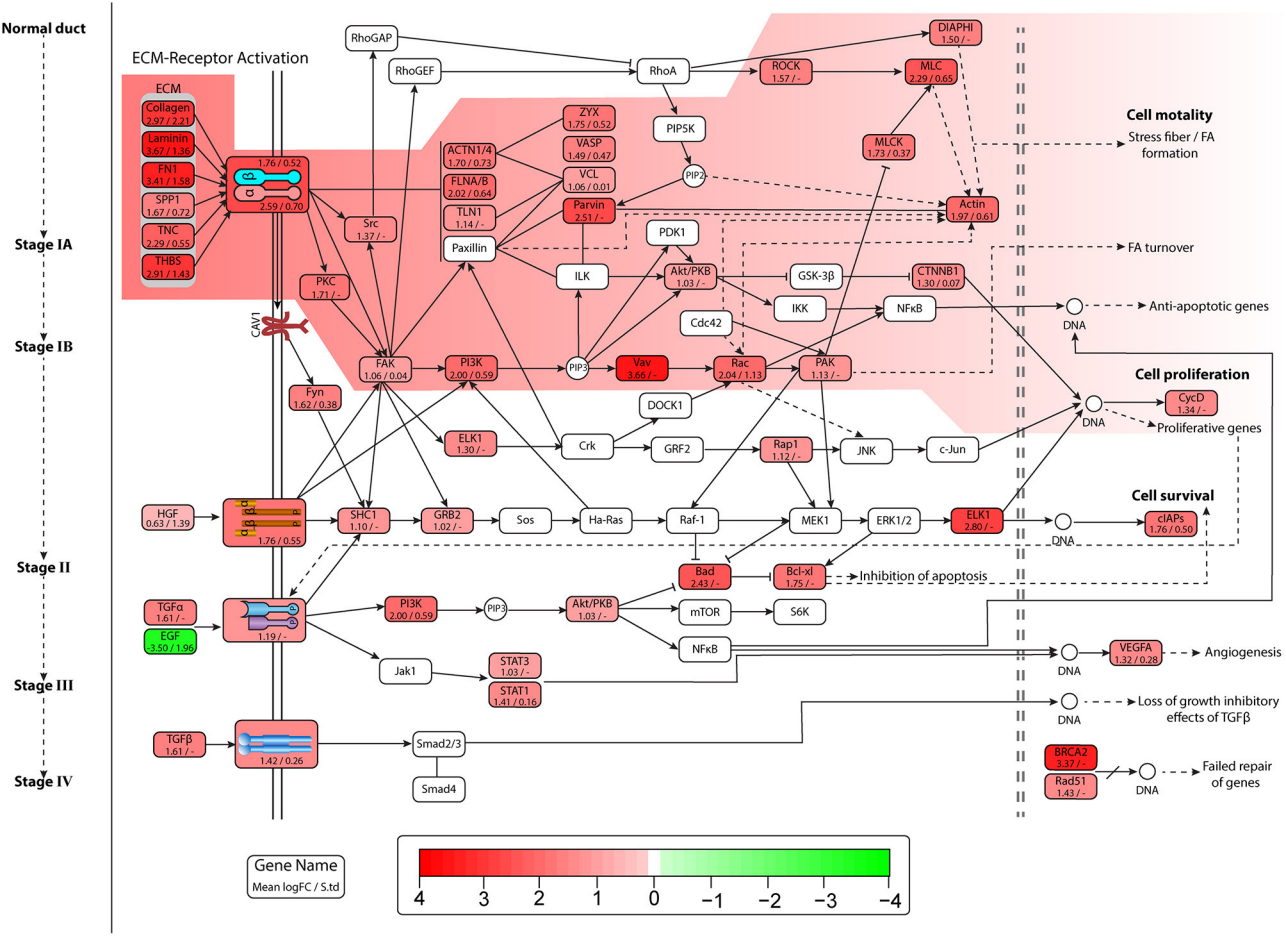
Figure represents expression of genes, and each entity contains name of gene mean *Log*_2_*FC* value and standard deviation of *Log*_2_*FC* values in all datasets. Pathway image is similar to Figure 3 depicting pancreatic cancer (PaCa) progression signaling in cell. Entities in the highlighted path show high overexpression, and lead to lamellipodia formation and initiation of proliferative gene expression.

**Figure 5:**
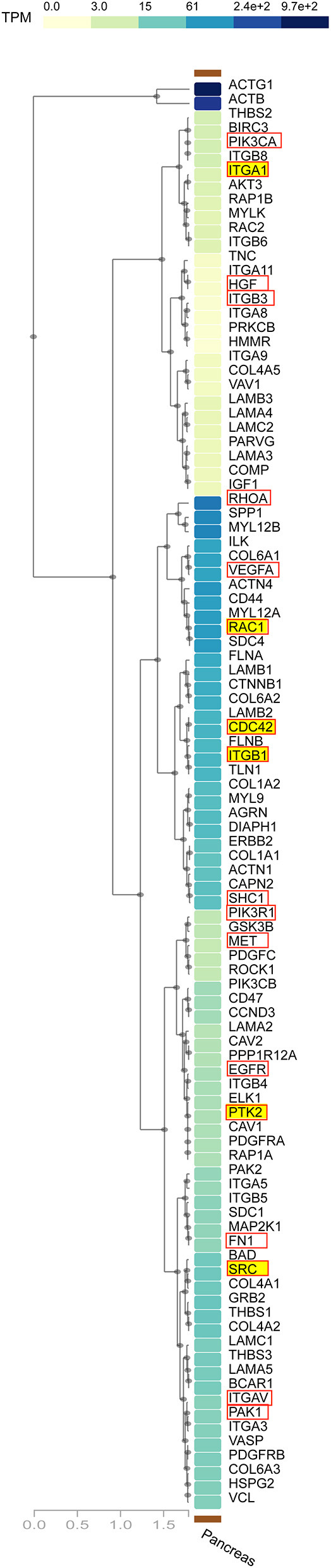
GTEx multigene expression graph. The expression profile of pancreatic cancer (PaCa)-related genes in normal pancreas. The names 6 Hub genes are highlighted with yellow color and of remaining hub genes are highlighted with red box. The graph represents that expression of hub genes are relatively low in normal pancreas tissues.

The ECM–receptor interaction and FA pathways were observed in all datasets, and were studied linking to the initiation and progression of PaCa. The detailed PaCa pathway integrating ECM–receptor interaction with FA pathways is given in Figures 3, 4. The GTEx normal pancreas expression data comparison of integrated pathway was also performed. To determine the difference in the expression of genes involve in PaCa tissue and normal pancreas cell. Figure 5 represents the gene expression values of normal pancreas in graphical representation, and values are provided in Supplementary Table 5. ECM molecules play important role in regulating the adhesion, motility, growth, and differentiation of cells. The integrin complexes are cell surface receptors, which are activated by ECM molecules and initiate multiple signaling cascades. The α and β subunits of integrin combine to form integrin complex. Different combination of α and β subunits co-expressing in PaCa samples are identified including ITGA1/ITGB1, ITGA3/ITGB1 ITGAV/ITGB3, ITGA2/ITGB1, and ITGA6/ITGB4. These co-expressed subunits also show overexpression in DE analysis. The mean *Log*_2_*FC* values of the α subunit ITGA1, ITGA2, ITGA3, ITGAV, and ITGA6 are 1.57, 2.60, 1.78, 1.30, and 1.56. The mean *Log*_2_*FC* values of β subunits ITGB1 and ITGB4 are 1.62 and 1.76, respectively. The expression value *Log*_2_*FC* of all subunits of integrin was less than 0.5 in GTEx data. According to this, the expression of integrins is high in PaCa than in normal pancreas. The ECM molecules include multiple proteins such as collagen, laminin subunit, secreted phosphoprotein 1 (SPP1), fibronectin1 (FN1), tenascin C (TNC), and thrombospondin (THBS). Collagen deposition is reported in PaCa extracellular environment, which results in the increase in rigidity of tumor. Multiple co-expressed collagen proteins are identified through WGCNA analysis, which are COL1A1, COL1A2, COL4A1, COL4A2, COL4A5, COL6A1, COL6A2, COL6A3, COL10A1, and COL12A1. All these co-expressed collagen proteins are also identified to be overexpressed in PaCa patients with *Log*_2_*FC* value >1.1. Most important types of collagen protein detected in all datasets with high fold change are COL10A1 and COL12A1. Mean *Log*_2_*FC* values of both are 2.97 and 2.56 correspondingly. The highly expressed ITGA2/ITGB1 integrin in PaCa is reported in multiple studies and collagen type I interaction with ITGA2/ITGB1 activates cellular pathways leading to the cell proliferation and migration (Grzesiak and Bouvet, 2006; Hamada and Masamune, 2018). The ITGA6/ITGB1 complex activated by ECM molecules also increases proliferation and migration by inducing ERK expression (Hamada and Masamune, 2018).

The laminins are extracellular matrix glycoproteins, which have 5 α, 4 β, and 3 γ chains. Elevated levels of LAMB3 and LAMC2 are associated with aggressiveness and motility of PaCa, and these can be used for prognosis (Yang et al., 2019). The co-expression of laminin subunit proteins such as LAMA2, LAMA3, LAMA4, LAMA5, LAMB1, LAMB2, LAMB3, LAMC1, and LAMC2 was also identified along with collagen proteins. All reported co-expressed laminins are also overexpressed, with the *Log*_2_*FC* value >1.1 (Supplementary Table 1). Most importantly LAMA3, LAMB3, and LAMC2 are identified in all datasets with means *Log*_2_*FC* values 2.38, 2.78, and 3.67, respectively. In normal pancreas (GTEx data), underexpression of LAMA3 and LAMB3 was determined with the *Log*_2_*FC* values −1.249 and −0.53, respectively. The fibronectin (FN) is a high-molecular weight (440 kDa) glycoprotein and have 20 variants. The high overexpression of FN1 was identified in 6 datasets (4 microarray, 2 RNA-seq) with mean *Log*_2_*FC* value of 3.41. The overexpressed SPP1 and TNC were also identified with mean *Log*_2_*FC* values of 1.67 and 2.29. The co-expression of THBS proteins such as THBS1, THBS2, and THBS3 was identified along with other ECM proteins. THBS1 and THBS2 are also identified as DEGs, overexpressed with mean *Log*_2_*FC* values 2.257068 and 2.910749, respectively (Supplementary Table 1).

All these ECM molecules interact with integrin complexes and activate multiple pathways. One of these is lamellipodia formation. The overexpressed genes of actin cytoskeleton regulation pathway lead to stress fiber (contractile actin bundles) and lamellipodia formation. The lamellipodia is a thin sheet like an actin protrusion form on the guiding side of a moving cell. The actin stress fiber is present in non-muscle cells and lead to cell motility. The actin regulators such as Rho-GTPases RhoA, cdc42, and Rac induce the formation of lamellipodia under the influence of extracellular stimuli (Anne, 2011). The important entities of the actin regulation pathway were reported to be co-expressed, and also show high overexpression in DE analysis, which is also shown in Figures 3, 4. The mean *Log*_2_*FC* values of Rac1 and Rac2 were 2.04 and 1.04, respectively. Rac1 notably expresses in very low quantity having *Log*_2_*FC* value 0.101 in normal pancreas. The RhoA downregulation results in decrease in lamellipodia formation (Anne, 2011).

Other pathways activated by different receptors are Akt/PKB, PI3k, and MAPK signaling pathways, which causes an increase in cell proliferation and cell survival (anti-apoptotic pathways). The integrin complex, MET and EGFR/HER2 receptors, activate the PI3K pathway, which leads to the expression of proliferative and anti-apoptotic genes, and also resulted in FA turnover. The focal adhesion kinase (FAK) also known as protein tyrosine kinase 2 (PTK2) activated by integrins and growth factor receptors is involved in multiple pathways. The overexpressed PTK2 was reported in two PaCa datasets, with mean *Log*_2_*FC* value of 1.06, while in normal pancreas expression was distinctly low *Log*_2_*FC* value 0.2. The growth factor receptors play very important role in initiating multiple oncogenic pathways. The MET and EGFR/HER2 were two receptors found highly overexpressed in our results along with their co-expression in PaCa samples. The MET receptor was reported in all datasets with mean *Log*_2_*FC* value of 1.76 and TCGA cohort expression value of 1.45. The overexpressed TGFβ1 and TGFBR1 were also identified, which induce the expression of genes causing loss of growth inhibitory effects of TGFβ. The TGFβ1 showed high expression level in six datasets with the mean *Log*_2_*FC* value of 1.79, while TGFBR1 is reported in three datasets with a value of 1.42.

**Figure 6:**
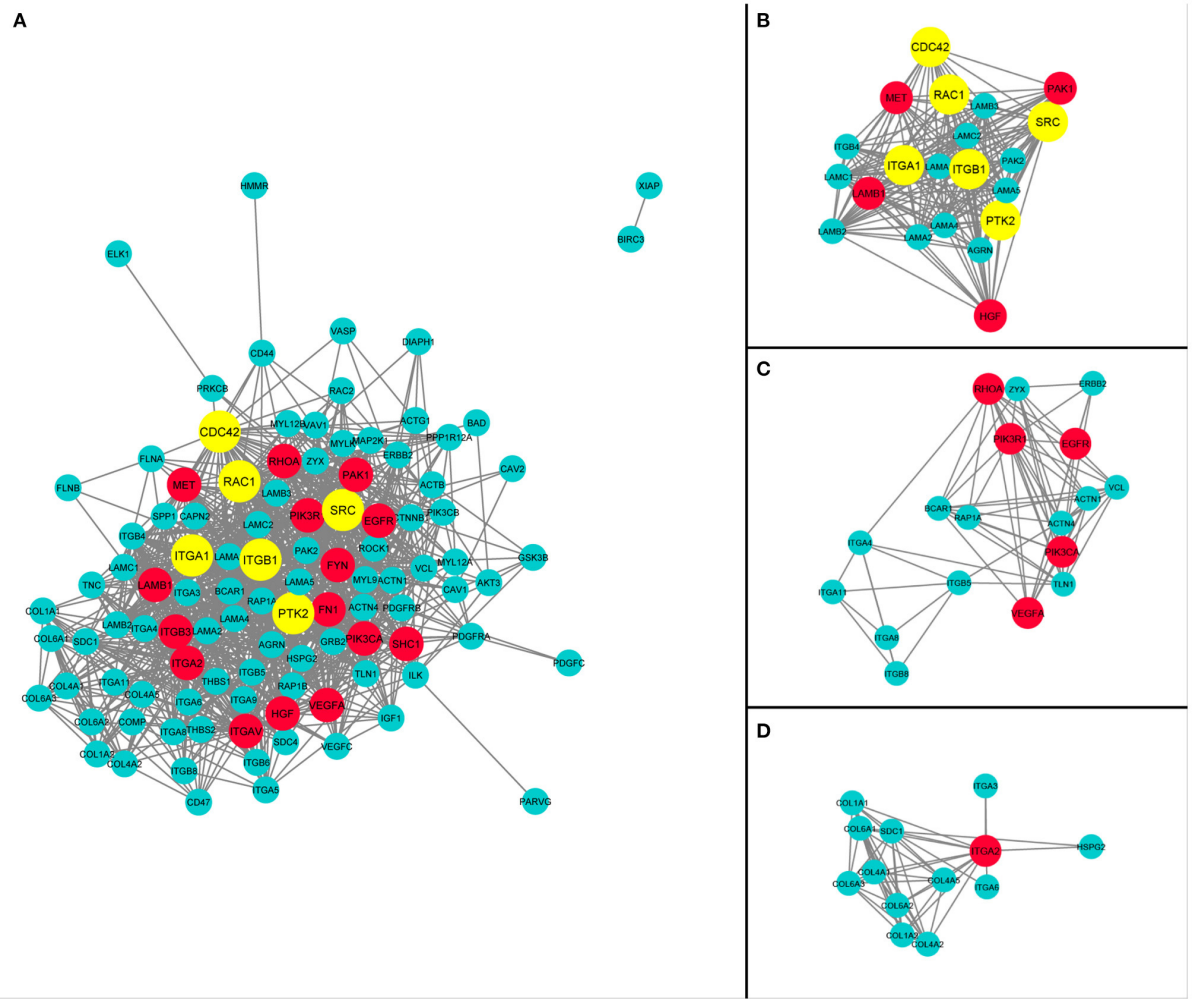
Hub genes of pancreatic cancer (PaCa) and protein–protein interaction network of entities given in Figures 3, 4. **(A)** The network indicates significant interactions in PaCa pathways. Fourteen hub genes highlighted with having highest interaction degree. Six Hub genes identified by CytoHubba app of Cytoscape are highlighted with yellow color. **(B)** PPI network of PaCa specifying 10 hub genes with a cluster of highly interactive genes in the network. **(C)** PPI network of PaCa specifying 5 hub genes with a cluster of second highly interactive genes in the network. **(D)** The cluster of third highly interactive genes with 1 hub genes in the network.

The protein–protein interaction network analysis of this integrated pathway was performed using STRING and Cytoscape to identify degree of interaction between all genes and determination of hub genes. The Network Analyze module of Cytoscape determine the statistics of network such as degree, betweenness, and closeness provided in Supplementary Table 6. The genes with higher number of interactions, like those with total degree ≥30, were considered as hub genes. A total of 20 hub genes were identified in this analysis. The red color coded entities in the network are hub genes, as given in Figure 6. The MCODE app of Cytoscape generated clusters containing genes with a high number of interactions. Three clusters were selected with cluster density score >8. The network diagram and most dense interacting cluster out of the whole network are provided in Figure 6. Afterward, six hub genes were identified by using the CytoHubba app. These genes are Cdc42, Rac1, ITGA1, ITGB1, SRC, and PTK2, and are shown by yellow color entities in Figure 6.

Apart from analyzing modules with high positive correlation, modules with high negative correlation (≤ −0.6) were also analyzed in context of PaCa development. Selected modules for all datasets are provided in Table 4. Significant hub genes with PaCa correlation < −0.6 and module membership (MM) value >0.6 were selected for all modules, provided in second data sheet (Supplementary Table 2). Then modules for each dataset were merged into one, and the enrichment of genes was performed using Enrichr. The Significant pathways with *p*-value < 0.05 were given in Supplementary Table 4, and the number of significant pathways (Table 4). To identify the most significant pathways retrieved in all datasets, common pathways were determined. The propanoate metabolism and calcium signaling pathway were identified as most significant, as all the predicted genes in these two pathways show underexpression. The calcium signaling pathway is crucial for the normal functioning of cells. Disruption of this pathway is reported in the early stages of multiple malignancies, along with PaCa (Gregório et al., 2020).

## 4. Discussion

PaCa is the most fatal malignancy due to its late prognosis. About 90% of the patients die in the first year of diagnosis, and almost all patients undergo metastases. For understanding the molecular nature and getting a deeper insight of regulatory pathways disrupted in PaCa cell, transcriptome analysis assists a lot. In this study, transcriptome analysis of PaCa cells in comparison to normal pancreatic cells is conducted. For this purpose, various microarray and RNA-seq datasets from different studies were collectively analyzed.

The first microarray data is E-GEOD-15471, in which study they had combined their results with previous studies. The t-test statistic was used for differential expression analysis, and determined that there is a strong correlation of keratin 7, laminin subunit γ 2 (LAMC2), stratifin (SFN), platelet phosphofructokinase (PFKP), annexin A2, MAP4K4, and MBOAT2 overexpression with patients death (Badea et al., 2008). All the genes reported in the above-mentioned study are also identified in our results, and show relatively high overexpression with the *Log*_2_*FC* value >1.

In the second microarray study (E-GEOD-28735), downregulation of DPEP1 (dipeptidase 1) was investigated. DPEP1 gene is involved in suppression of cell proliferation and cancer invasiveness. EGF signaling pathway regulates the expression of DPEP1 (Zhang et al., 2012). In our analysis, downregulated DPEP1, along with TXP2 upregulation, is detected. The TXP2 gene shows oncogenic properties, and its overexpression results in the poor patient survival.

In the third microarray study, miRNAs target against different mRNAs were analyzed, and explored the macrophage migration inhibitory factor (MIF) pathway. The study determined that upregulated MIF trigger miR-301b overexpression. The overexpressed miR-301b suppresses the expression of orphan nuclear receptor NR3C2. The NR3C2 normal expression is important for cell survival (Yang et al., 2016). In our analysis, we only included mRNA samples submitted with accession number (E-GEOD-62452). Our results showed concordance with the output of the above-mentioned study. We predicted downregulated NR3C2 with the mean *Log*_2_*FC* value of −1.50. The overexpression of various cellular adhesion (collagen proteins and FN1) and interaction receptors (integrins and MET) are detected in differential gene expression analysis.

The fourth microarray study comprises identifying mRNA regulation by miRNAs (E-GEOD-41368). They determined that three miRNAs including MIR21, MIR23A, and MIR27A suppress the expression of 3 tumor suppressor genes including programmed cell death 4 (PDCD4), BTG anti-proliferation factor 2 (BTG2), and neural precursor cell expressed/developmentally down-regulated 4-like (NEDD4L), respectively (Frampton et al., 2014). In our results, downregulation of these three tumor suppressor genes was determined with the mean *Log*_2_*FC* values < −1.5.

In the fifth microarray study (E-GEOD-18670), the differentially expressed genes in cancer cell responsible for metastases development in PDAC patients were inquired. For this purpose, the expression level of tumor cells was compared with non-tumoral pancreatic cells, circulating tumor cells, and haematological cells. They proposed that six highly overexpressed genes C19orf33, ECT2, IL1RN, S100P, SFN, and TUBA4A in PDAC cells are responsible of cell motility and invasiveness (Sergeant et al., 2012). All these genes are detected as highly overexpressed in our analysis, along with other 798 overexpressed genes. Most of the predicted genes involved in the pathways of cancer. The genes C19orf33, S100P, and SFN show high expression with the *Log*_2_*FC* values of 2.87, 3.04, and 2.72, respectively.

Two RNA-seq datasets were also analyzed for this study. The first RNA-seq study comprises integrated network determination of miRNAs and mRNAs in PaCa. They used TopHat2 tool for sequence alignment, and DESeq2 for differential gene expression analysis. For determining miRNA and mRNA relationship, Human miRNA Disease Database (HMDD) and miR2Disease database were used (Lin et al., 2019). In our analysis, only mRNA sample files are processed for DEGs identification, sequence files are aligned using HISAT2 tool, and DESeq2 is used for differential gene expression analysis. Our results are similar to the above study for significant genes identification. The second RNA-seq study comprises total RNA content included coding (mRNAs) and non-coding (miRNAs, snoRNAs, snRNAs, and pseudogenes) (Müller et al., 2015). In our study, we only analyzed these data for identification of mRNAs expression.

From the comparative analysis of DEGs, 76 significant genes are identified, which are further interrogated for involvement in PaCa-related pathways. Seven genes were downregulated, out of these seven genes, AOX1, C5, and IAPP are involved in crucial pathways for normal functioning of cells. The most important of all underexpessed genes is aldehyde oxidase 1 (AOX1) gene, detected with mean *Log*_2_*FC* value of −2.44. It belongs to molybdenum hydroxylase family, it oxidizes aldehydes, a toxic metabolic product of ethanol and heterocyclic rings, and produces reactive oxygen species. It requires metal molybdenum and the flavin cofactor for this catalytic reaction. AOX1 helps as well in the production of retinoic acid, which triggers the signaling pathway important for pancreas development and normal functioning. High expression levels of AXO1 were predicted in normal pancreatic cells, whereas low expression to zero expression is identified with respect to PaCa stages in an immunohistochemical study (Crnogorac-Jurcevic et al., 2005).

Remaining 69 genes are upregulated and are involved in different pathological pathways. Some of the upregulated genes are tumorigenic, which facilitates hypoxia, cell proliferation, invasion, and metastasis. Overexpressed cell surface molecules identified are CEACAM1, CEACAM6, FXYD3, MET, ADGRF1, and transmembrane protease serine 4 (TMPRSS4). The carcinoembryonic antigen-related cell adhesion molecule 6 (CEACAM6) induces epithelial-mesenchymal transition (EMT), which causes tumor cell invasion in PaCa patients (Chen J. et al., 2013). CEACAM1 and CEACAM6 increase neutrophil degranulation, and their elevated expression level is a useful indicator of PaCa development in body (Simeone et al., 2007; Chen J. et al., 2013). The FXYD domain containing ion transport regulator 3 (FXYD3) is the member of the small membrane proteins family. FXYD3 function as Na/K-ATPase regulator and induces a hyperpolarization-activated current in membrane by changing K+ and Na+ affinity of Na/K-ATPase (Bibert et al., 2006; Peron et al., 2019). FXYD3 is also known as mammary tumor marker 8 (Mat-8), and cell proliferation is considered as a plausible role of FXYD3 in cancer. In a previous study, 3.4-fold increase in FXYD3 was identified in 50% of PDAC samples with strong levels of significance (Kayed et al., 2006). High mean *Log*_2_*FC* of 2.14 is determined in our study with its overexpression in all datasets, along with TCGA cohort result (3.07).

Adhesion G protein-coupled receptor F1 (ADGRF1) is also known as G protein-coupled receptor 110 (GPR110) encoded by GPR110 gene. It belongs to the largest family of cell membrane proteins G protein-coupled receptors (GPCRs). Cellular pathways activated by GPR110 and its physiological function are still unknown. However, its expression is correlated with the cellular malignancy, cancer invasion, and metastases (Lum et al., 2010; Sahay et al., 2016; Sadras et al., 2017). Strong correlation of GPR110 gene with PaCa was identified in a study where its overexpression is observed in all PaCa patients (Lin et al., 2019). The solute carrier family 2 member 1 (SLC2A1) gene code for glucose transporter 1 (or GLUT1) protein facilitates the transport of glucose across plasma membranes. SLC2A1 is predicted to be involved in central carbon metabolism in cancer, HIF-1 signaling pathway, and adipocytokine signaling pathway. All these pathways collectively are involved in the development and progression of cancer by increasing cell proliferation and evading apoptosis.

Along with the individual results of DE analysis, co-expression network analysis results were combined with DE results in this study and TCGA cohort was used for validating gene expression. The ECM–receptor interaction and FA pathways predicted through co-expression analysis were reported to be involved in PaCa development and progression. All the predicted pathways were studied conjointly, regarding the gradual progression of PaCa from stage IA to the metastatic phase (Figures 3, 4). Moreover, the protein–protein interaction network analysis of pathways was performed to determine the putative PaCa biomarkers/ hub genes. The integrins interacting with extracellular matrix and growth factor receptors initiate multiple pathways, which causes the development and aggressiveness of PaCa. The overexpressed integrin α (ITGA) subfamily genes ITGA2 and ITGA3 are predicted to be involved in cancer-related pathways such as PI3K-Akt signaling pathway and FA interacting with ECM genes such as laminin (LAMB3, LAMA3, and LAMC2) and collagen (COL10A1, COL12A1) (Grzesiak and Bouvet, 2006; Hamada and Masamune, 2018; Yang et al., 2019).

The ITGA1 and ITGB1 are considered as early-stage biomarkers of PaCa, which increase the invasiveness. Both ITGA1 and ITGB1 are identified as hub genes in network analysis (Figure 6). ITGA1 in combination with collagen also promotes gemcitabine therapy resistance by inducing overexpression of TGFβ (Gharibi et al., 2017). The overexpression of ITGA2 plays an essential role in tumor progression, metastasis, and motility. ITG2 activates upregulate STAT3 signaling pathway, which resulted in tumor progression (Ren et al., 2019).

The other identified biomarkers/hub genes on the basis of network analysis are MET, PTK2, Rac1, SRC, and LAMB1 (Figure 6). The MET proto-oncogene is receptor tyrosine kinase (MET), also known as hepatocyte growth factor receptor (HGFR). It is a multi-domain receptor consisted of an extracellular semi domain, cysteine-rich Met-related sequence (MRS), glycine–proline-rich (G-P) repeats, and four immunoglobuline-like structures, attached to intracellular regions including tyrosine kinase domain (Gentile et al., 2008). MET is expressed as single-chain precursor, which then cleaved into α and β subunits. Growth factor HGF/SF binds and activates MET, normal activation induces embryogenesis, while abnormal activation of MET in cancer leads rapid progression by activating multiple signaling pathways including Ras, PI3K, STAT, Wnt, and Notch signaling pathway. Ras signaling activates MAPK and causes cell proliferation, while other pathways induce cell invasion, metastatic growth, and angiogenesis (Abounader et al., 2004; Gentile et al., 2008). MET is considered as biomarker for the identification of PaCa and a promising therapeutic target (Li et al., 2011). The MET protein overexpression in cell is associated with the activation of multiple cancer-related pathways such as PI3K-Akt signaling pathway, FA, and central carbon metabolism in cancer pathways. The FAK/PTK2 is kinase protein play crucial role in multiple cancer-related pathways activated by various surface proteins integrin and growth factor receptor. It also promotes p53 degradation through ubiquitination in nucleus (Zhou et al., 2019). The RAC is downstream protein involving in pathways for the formation of lamellipodia under the influence of extracellular stimuli and activation of the proliferative genes. In normal pancreas, RAC expression is precisely low, as the main function of RAC is to activate the formation of actin fiber in normal cells. In PaCa cells, its overexpression leads to abundant deposition of actin and lamellipodia formation (Anne, 2011).

## 5. Conclusions

PaCa is a fatal malignancy with 5-year survival rate <7 and >98% metastasis development rate. There is a dire need for developing new therapies for reducing mortality rates. For attaining this, it is essential to thoroughly study pathways and genes involved in initiation, progression, and invasiveness of PaCa. The whole transciptome analysis along with co-expression network analysis of PaCa provides the prime way to explore entities that are differentially co-expressed in the system. Most important hub genes and pathways determined in this study are integrins (ITGA1, ITGB1), LAMA1, MET, FAK, and Rac1-mediated actin bundle deposition, PI3k/Akt, and MAPk signaling pathways. The STAT3 activation is induced by integrin, VEGFA, and EGFR, which leads to angiogenesis and loss of growth inhibitory effects of TGFβ induced by TGFB1 and TGFβR1/TGFβR2. The hub genes and overexpressed surface receptors initiating all these pathways can be used for designing advance therapy against these receptors.

## Data Availability Statement

The datasets presented in this study can be found in online repositories. The names of the repository/repositories and accession number(s) can be found in the article/Supplementary Material.

## Author Contributions

MN and RP conceived and designed the study. MN conducted the analysis and wrote the manuscript. SZ helps in the conducting of network analysis part of methodology. All authors took part in analytical discussions and critical reviewing of manuscript.

## Conflict of Interest

The authors declare that the research was conducted in the absence of any commercial or financial relationships that could be construed as a potential conflict of interest.
