# Supplementary material for: Integrated Analysis of Microarray and RNA-Seq Data for the Identification of Hub Genes and Networks Involved in the Pancreatic Cancer
Source: Front Genet. 2021 Jun 23;12:663787. doi: 10.3389/fgene.2021.663787 (PMC8273913; doi:10.3389/fgene.2021.663787)
Supplement: Supplementary file 1 [file Data_Sheet_1.pdf]

Supplementary Material

1 VOLCANO PLOTS OF ALL DATASETS:

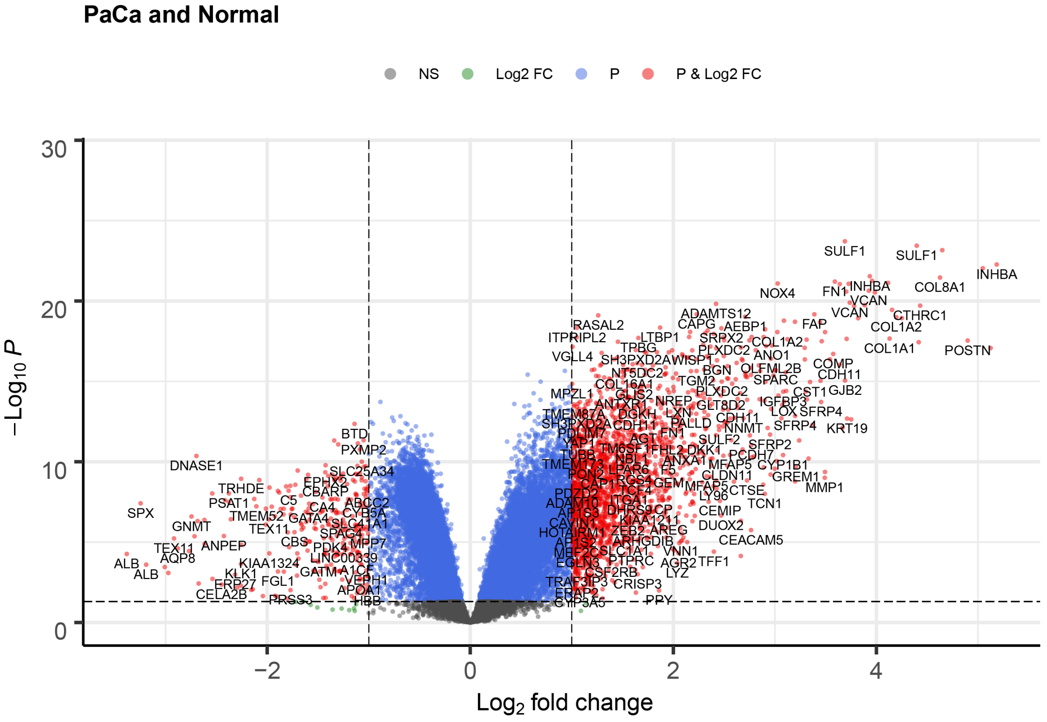

Figure (S1) **Volcano plot of DEGs for E-GEOD-15471 dataset** The red dots in upper left corner of graph represents underexpressed genes with p-value less than 0.05 ($-Log_{10}P$ value greater than 1.3) and fold change less than -1. Red dots in the upper right corner of graph represents overexpressed genes with p-value less than 0.05 ($-Log_{10}P$ value greater than 1.3) and fold change greater than 1. The overall 1665 DEGs retrieved from dataset E-GEOD-15471 full filling log_2FC and p-value criteria, out of which 1466 genes were overexpressed while 199 genes were underexpressed. The list of DEGs with log_2FC values is provided in Supplementary Table 1.

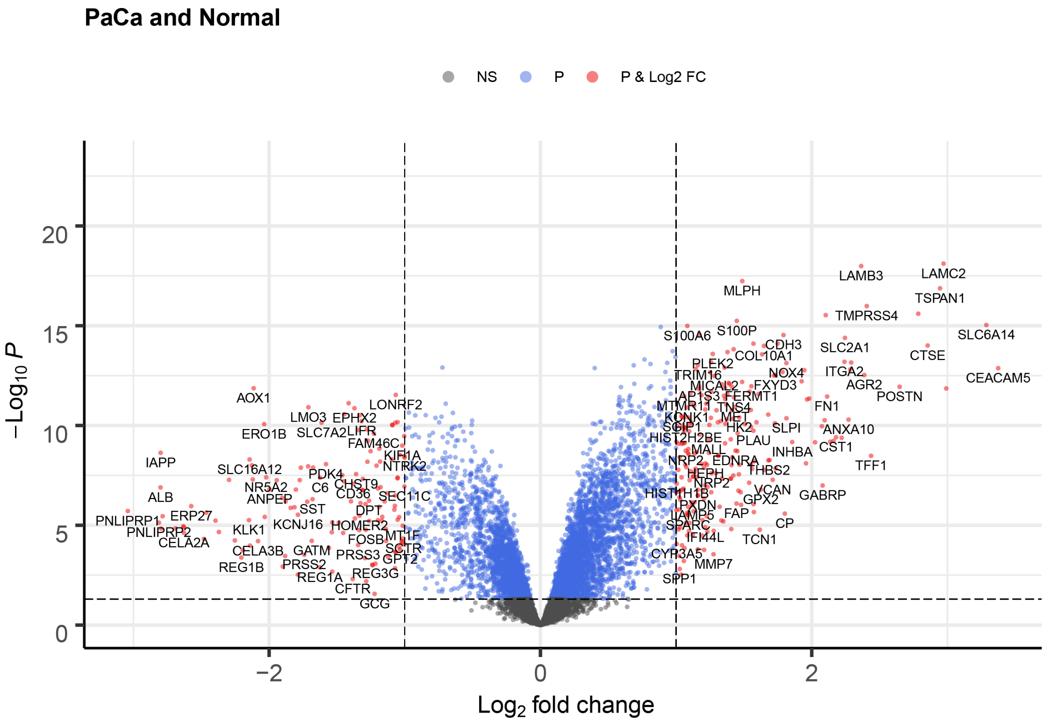

Figure (S2) **Volcano plot of DEGs for dataset E-GEOD-28735** After p-value and log2FC threshold filtering 364 DEGs were identified, with 227 overexpressed and 137 underexpressed genes. The list of DEGs along with log_2FC values is provided in Supplementary Table 1.

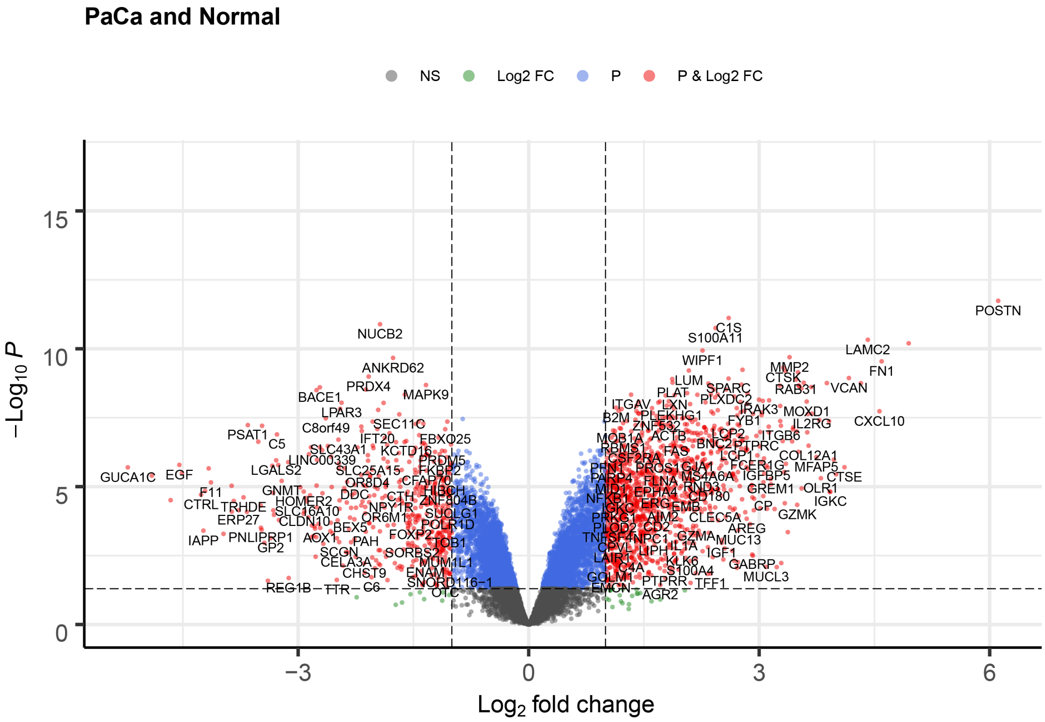

Figure (S3) **Volcano plot of DEGs for dataset E-GEOD-41368** The overall 1688 significant DEGs were retrieved, out of which 1222 overexpressed and 466 were underexpressed genes. The list of DEGs along with log_2FC values is provided in Supplementary Table 1.

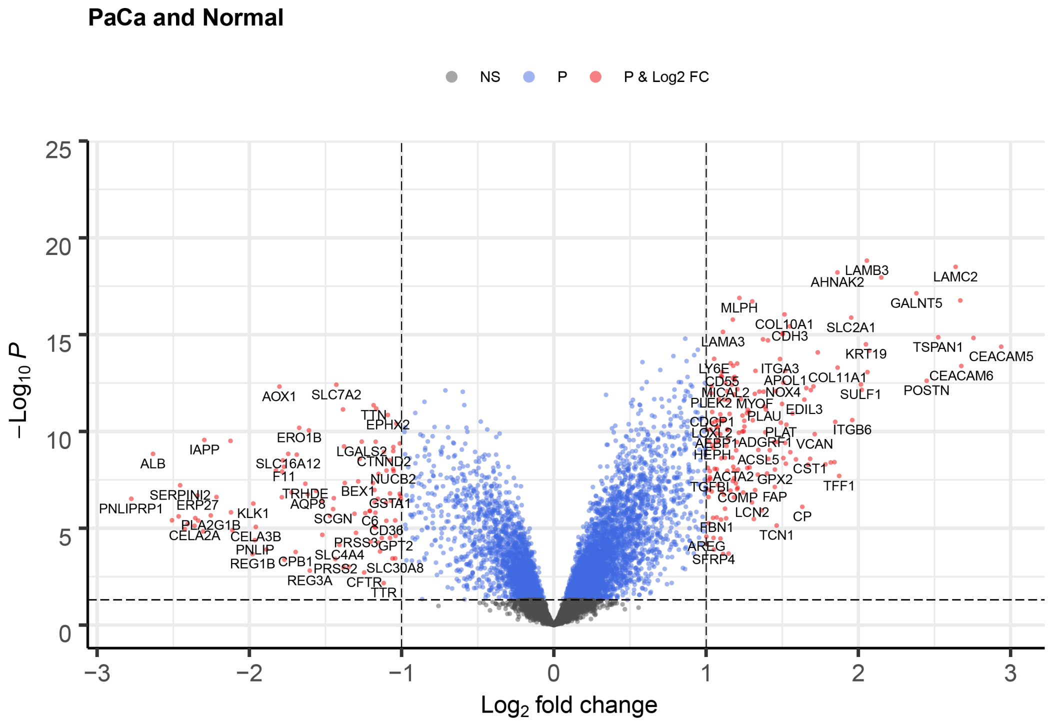

Figure (S4) **Volcano plot of DEGs for dataset E-GEOD-62452** The total 274 DEGs obtained (174 overexpressed and 100 underexpressed), The list of DEGs along with the log_2FC values is provided in Supplementary Table 1.

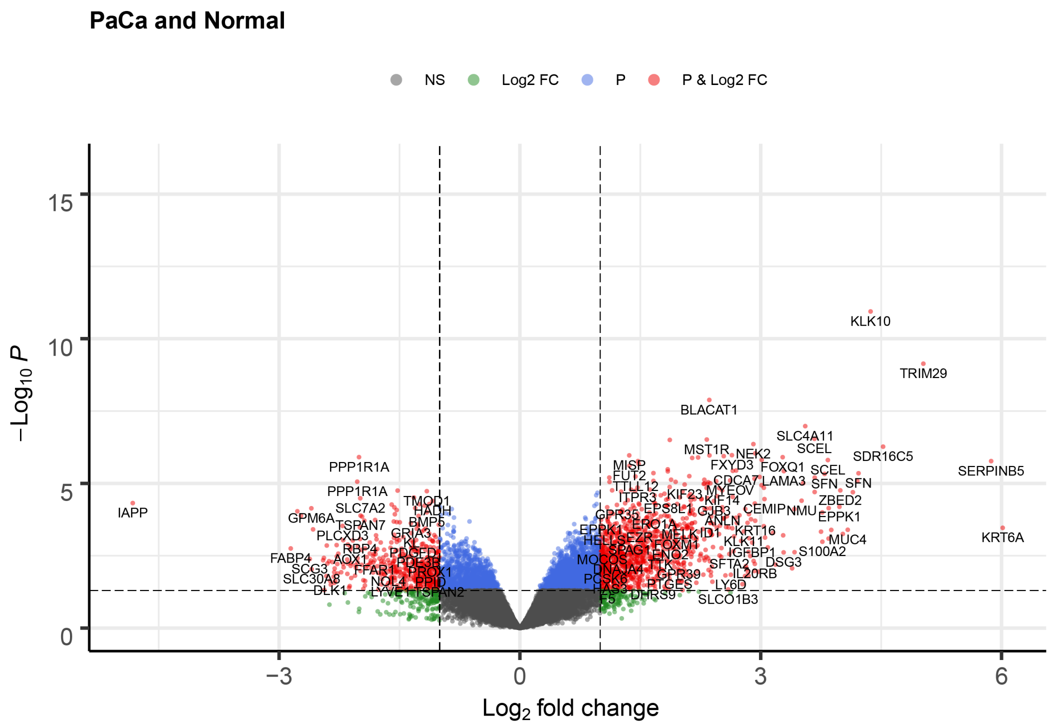

Figure (S5) **Volcano plot of DEGs for dataset E-GEOD-18670**For E-GEOD-18670 total number of DEGs obtained after differential expression analysis were 1164, with 798 overexpressed and 366 underexpressed genes, The lists of DEGs with their log_2FC values are provided in Supplementary Table 1.

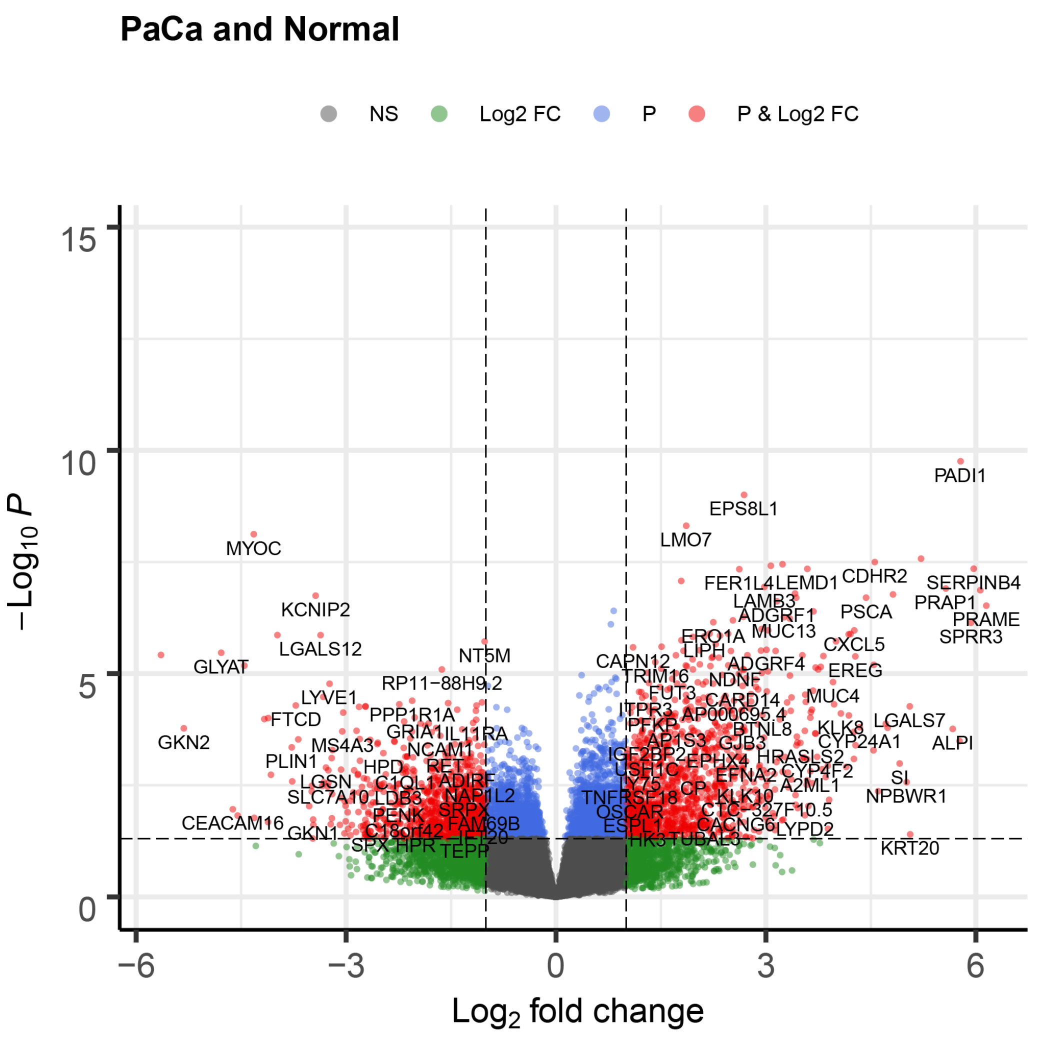

Figure (S6) **Volcano plot of DEGs for dataset GSE119794** Total of 2174 DEGs were retrieved (1194 up-regulated and 980 down-regulated). The list of DEGs with the log_2FC values is provided in Supplementary Table 1.

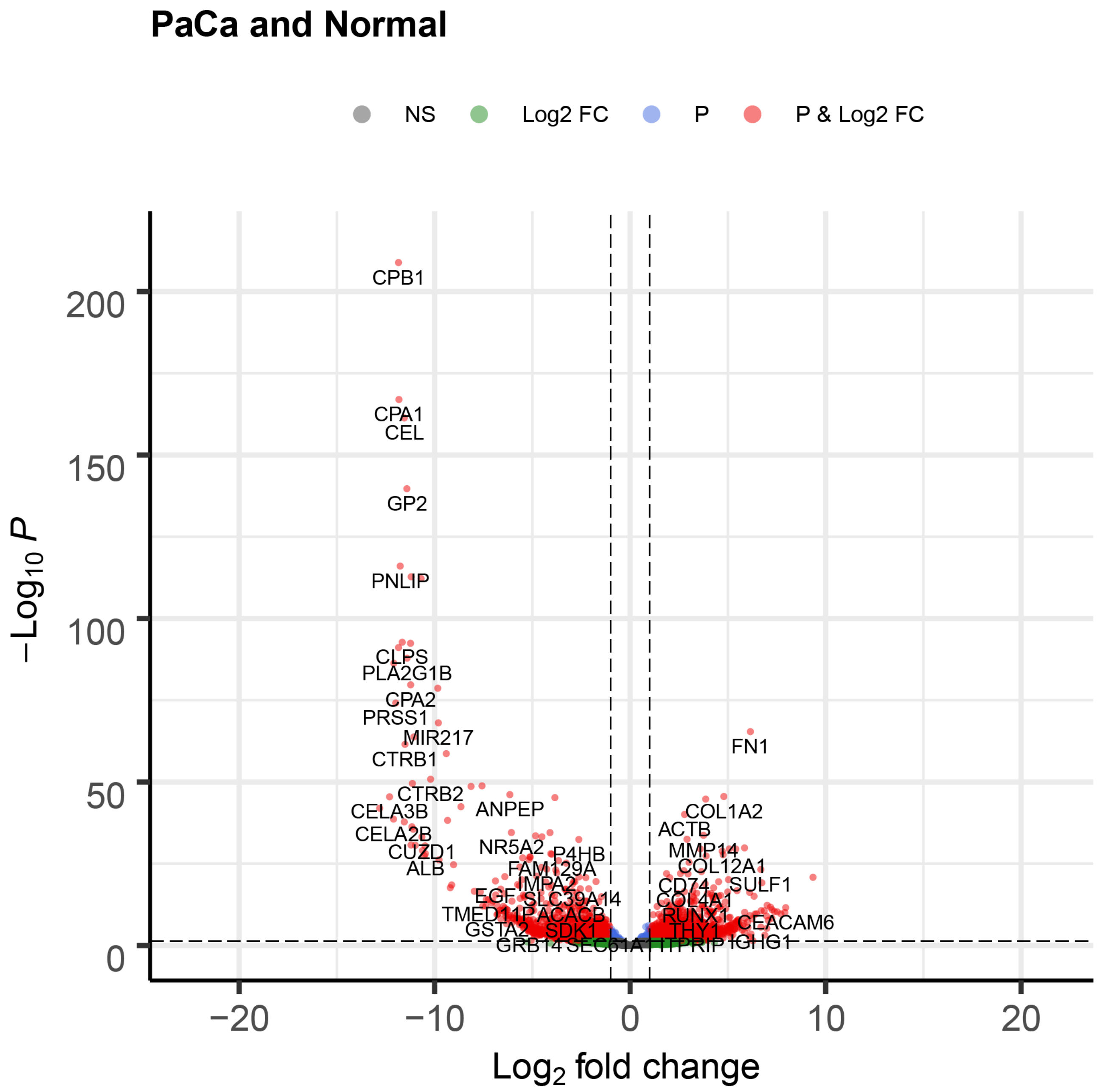

Figure (S7) **Volcano plot of DEGs for dataset E-GEOD-3494** Total 3804 DEGs identified, out of which 2193 gene were up-regulated and 1611 genes were down-regulated. The list of DEGs with the log_2FC values is provided in Supplementary Table 1.

2 WGCNA SOFT THRESHOLDS AND FINAL MODULES OF ALL DATASETS:

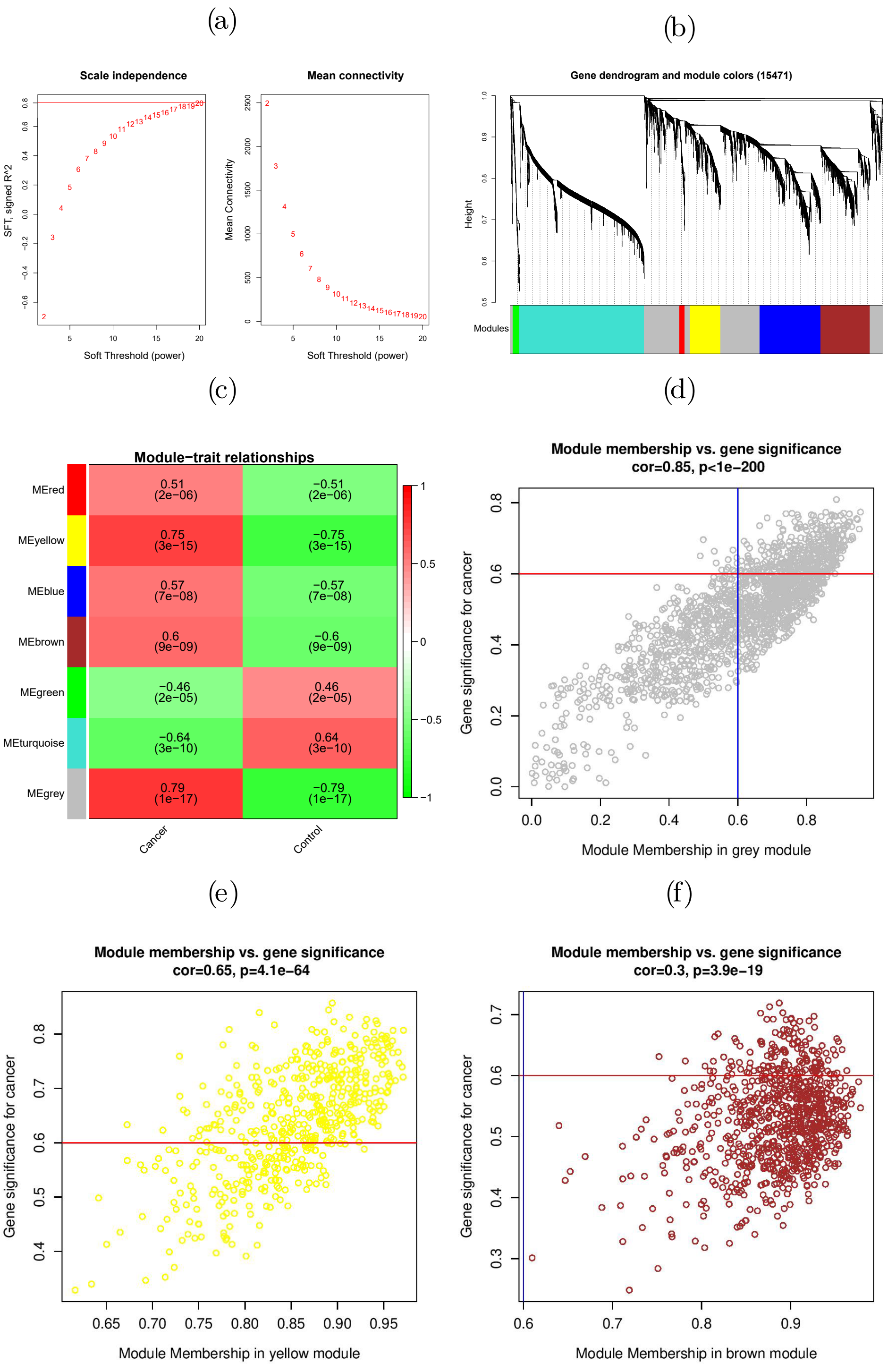

Figure (S8) Plots of Dataset E-GEOD-15471 **(a)** Scale Free Topology (SFT) model fitting construct scale-free network. Soft threshold value (β) from 2 to 20 was evaluated to obtain scale-free network on $R^2 \geq 0.8$. The β of 20 was determined on the basis of scale independence plot. **(b)** Gene tree (dendogram) along with final modules with module colors. Total 7 modules were formed on β value of 20. **(c)** Modules correlation plot with pancreatic cancer and control, along with correlation values and p-values for all modules. **(d)** Scatter plot for module membership vs. gene significance for PC in the grey module. Hub genes with significance for cancer and Module membership > 0.6 were selected for further analysis. **(e)** Scatter plot for module membership vs. gene significance for PC in the yellow module. **(f)** Scatter plot for module membership vs. gene significance for PC in the brown module.

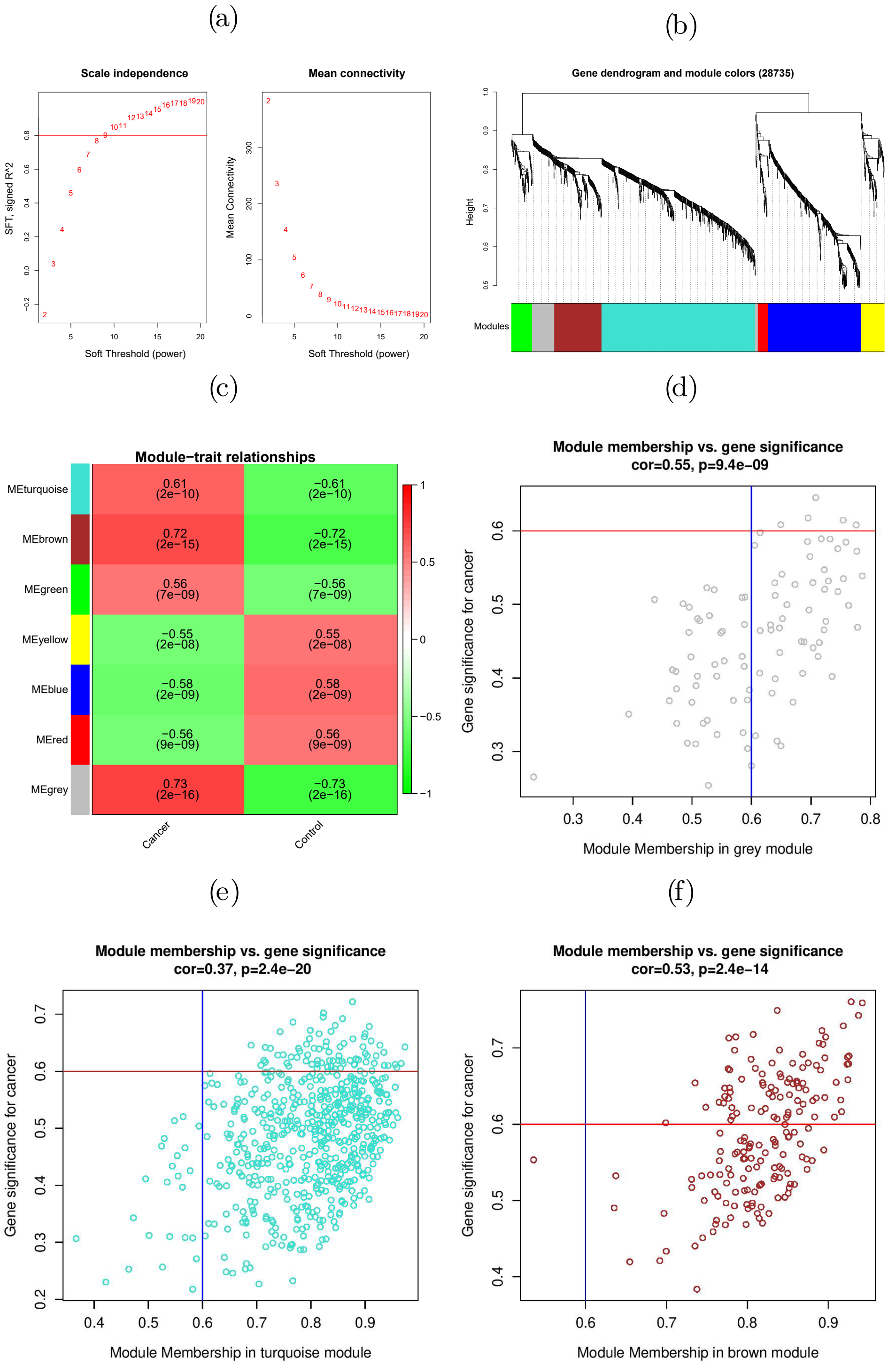

Figure (S9) Plots of Dataset E-GEOD-28735 **(a)** β value from 2 to 20 was evaluated to obtain scale-free network on $R^2 \geq 0.8$. The β of 9 was determined on the basis of scale independence plot. **(b)** Gene tree (dendogram) along with final modules with module colors. Total 7 modules were formed on β of 9. **(c)** Modules correlation plot with pancreatic cancer and control, along with correlation values and p-values for all modules. **(d)** Scatter plot for module membership vs. gene significance for PC in the grey module. **(e)** Scatter plot for module membership vs. gene significance for PC in the turquoise module. **(f)** Scatter plot for module membership vs. gene significance for PC in the brown module.

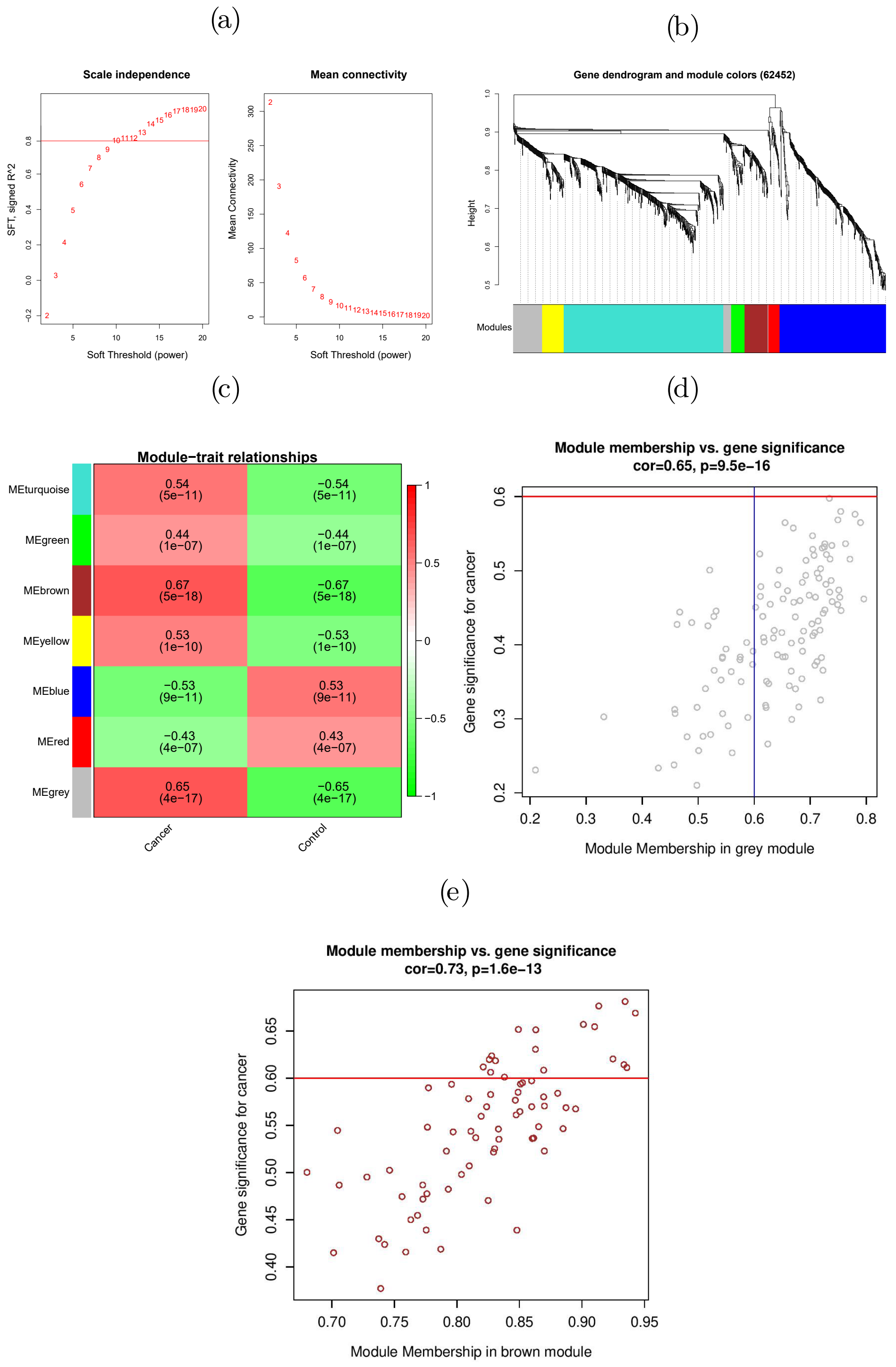

Figure (S10) Plots of Data E-GEOD-62452 **(a)** β value from 2 to 20 was evaluated to obtain scale-free network on $R^2 \geq 0.8$. The β of 10 was determined on the basis of scale independence plot. **(b)** Gene tree (dendogram) along with final modules with module colors. Total 7 modules were formed on SFT of 10. **(c)** Modules correlation plot with pancreatic cancer and control, along with correlation values and p-values for all modules. **(d)** Scatter plot for module membership vs. gene significance for PC in the grey module. **(e)** Scatter plot for module membership vs. gene significance for PC in the brown module.

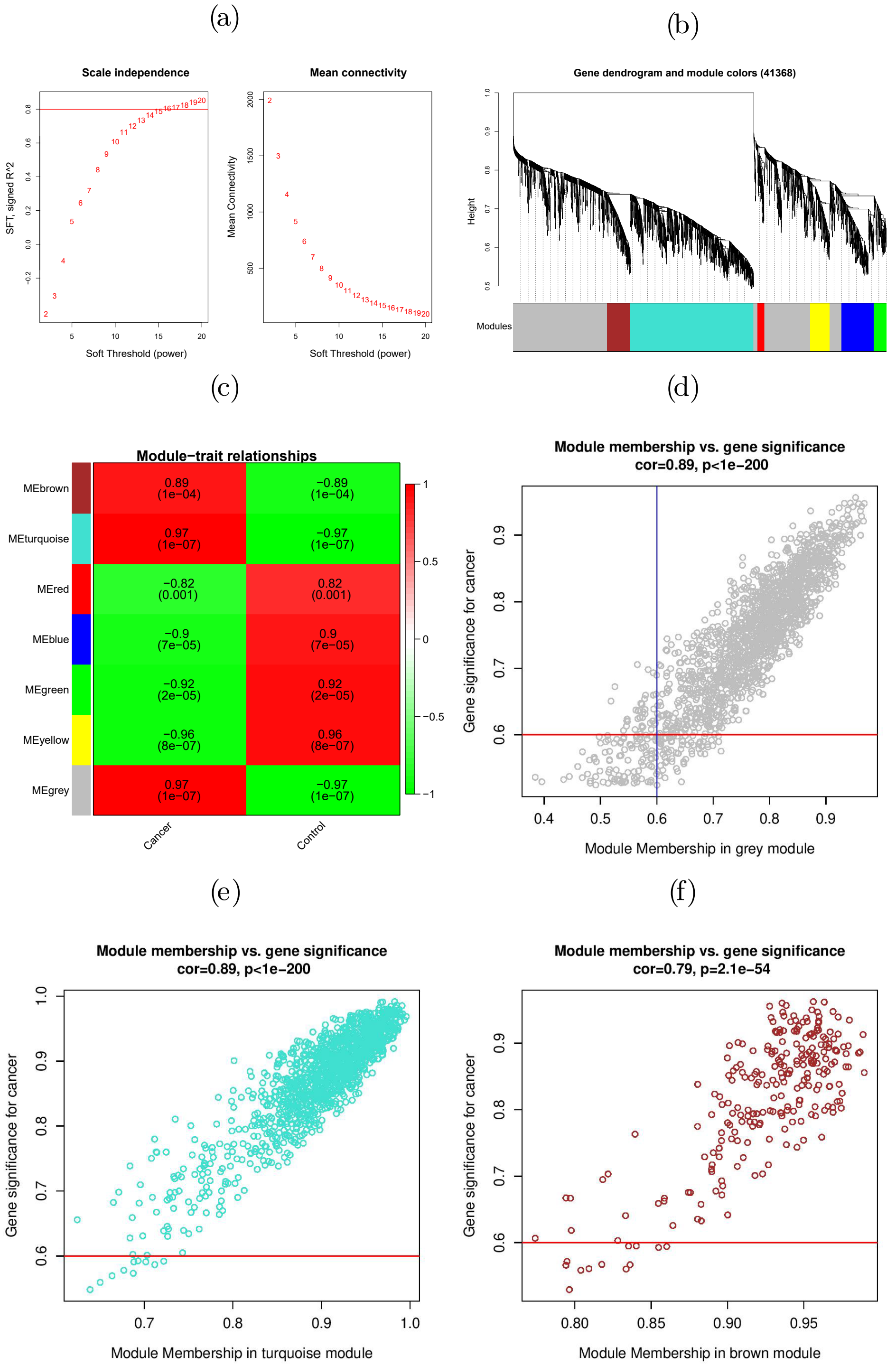

Figure (S11) Plots of E-GEOD-41368 **(a)** β value from 2 to 20 was evaluated to obtain scale-free network on $R^2 \geq 0.8$. The β of 16 was determined on the basis of scale independence plot. **(b)** Gene tree (dendogram) along with final modules with module colors. Total 7 modules were formed on SFT of 16. **(c)** Modules correlation plot with pancreatic cancer and control, along with correlation values and p-values for all modules. **(d)** Scatter plot for module membership vs. gene significance for PC in the grey module. **(e)** Scatter plot for module membership vs. gene significance for PC in the turquoise module. **(f)** Scatter plot for module membership vs. gene significance for PC in the brown module.

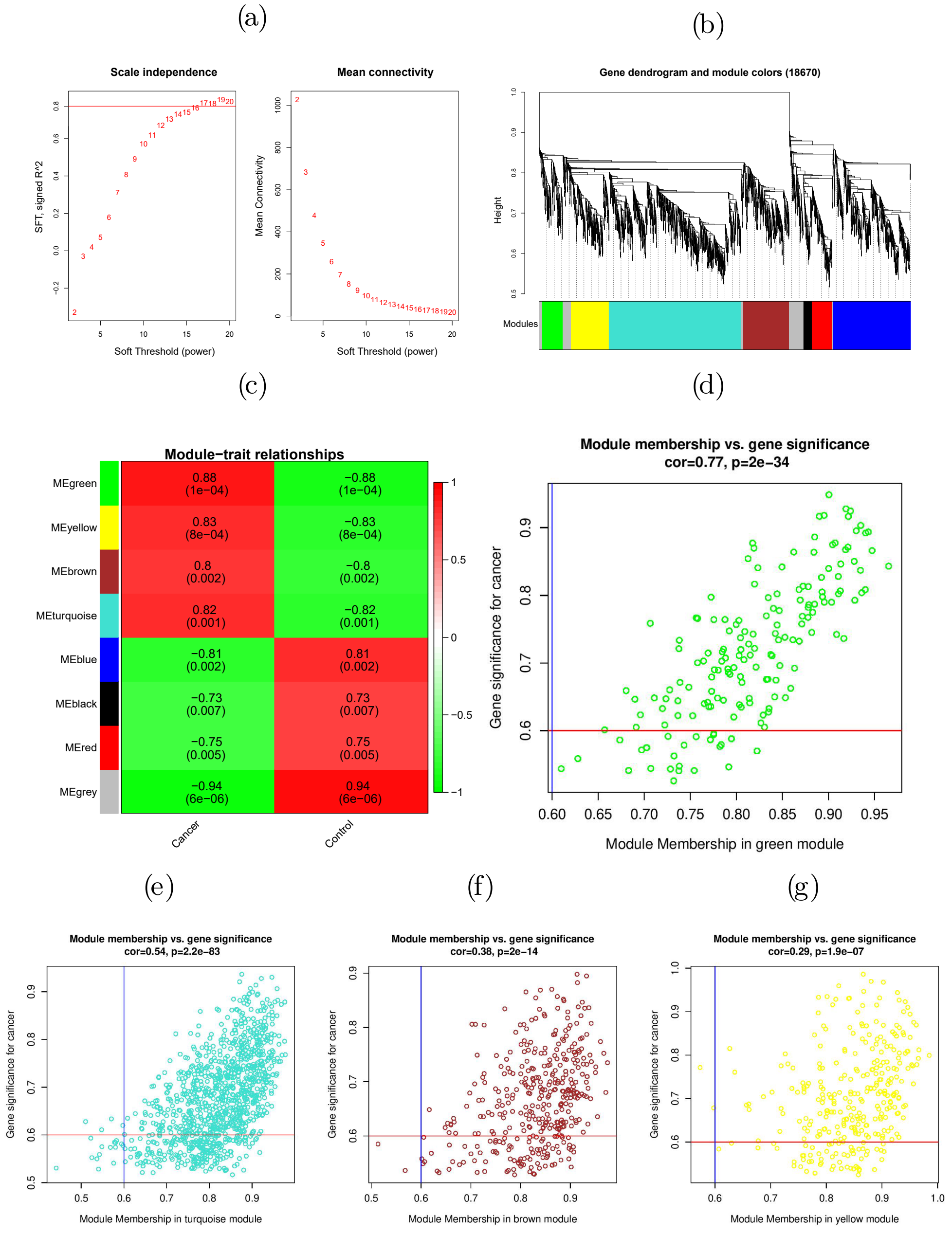

Figure (S12) Plots of Data E-GEOD-18670 **(a)** β value from 2 to 20 was evaluated to obtain scale-free network on $R^2 \geq 0.8$. The β of 16 was determined on the basis of scale independence plot. **(b)** Gene tree (dendogram) along with final modules with module colors. Total 8 modules were formed on SFT of 16. **(c)** Modules correlation plot with pancreatic cancer and control, along with correlation values and p-values for all modules. **(d)** Scatter plot for module membership vs. gene significance for PC in the green module. **(e)** Scatter plot for module membership vs. gene significance for PC in the turquoise module. **(f)** Scatter plot for module membership vs. gene significance for PC in the brown module. **(g)** Scatter plot for module membership vs. gene significance for PC in the yellow module.

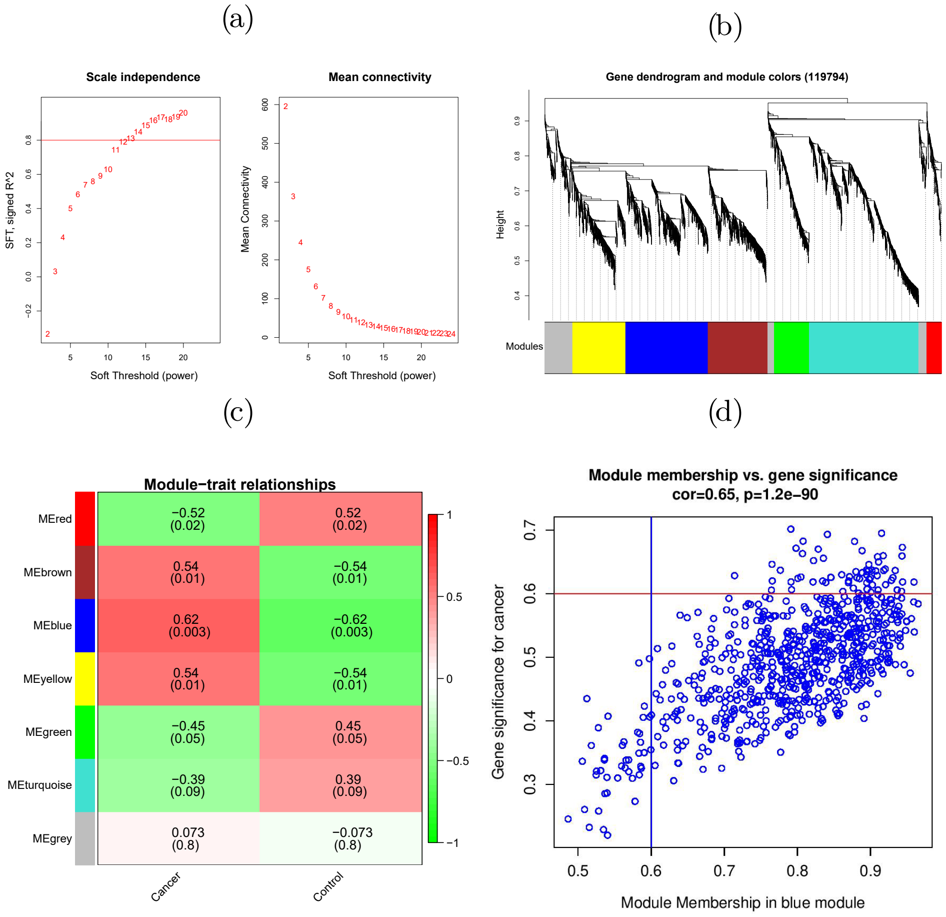

Figure (S13) Plots of Dataset GSE119794 (a) β value from 2 to 20 was evaluated to obtain scale-free network on $R^2 \geq 0.8$. The β of 12 was determined on the basis of scale independence. (b) Gene tree (dendogram) along with final modules colors, Total 7 modules were formed on SFT of 12. (c) Modules correlation plot with pancreatic cancer and control, along with correlation values and p-values for all modules. (d) Scatter plot for module membership vs. gene significance for PC in the blue module.

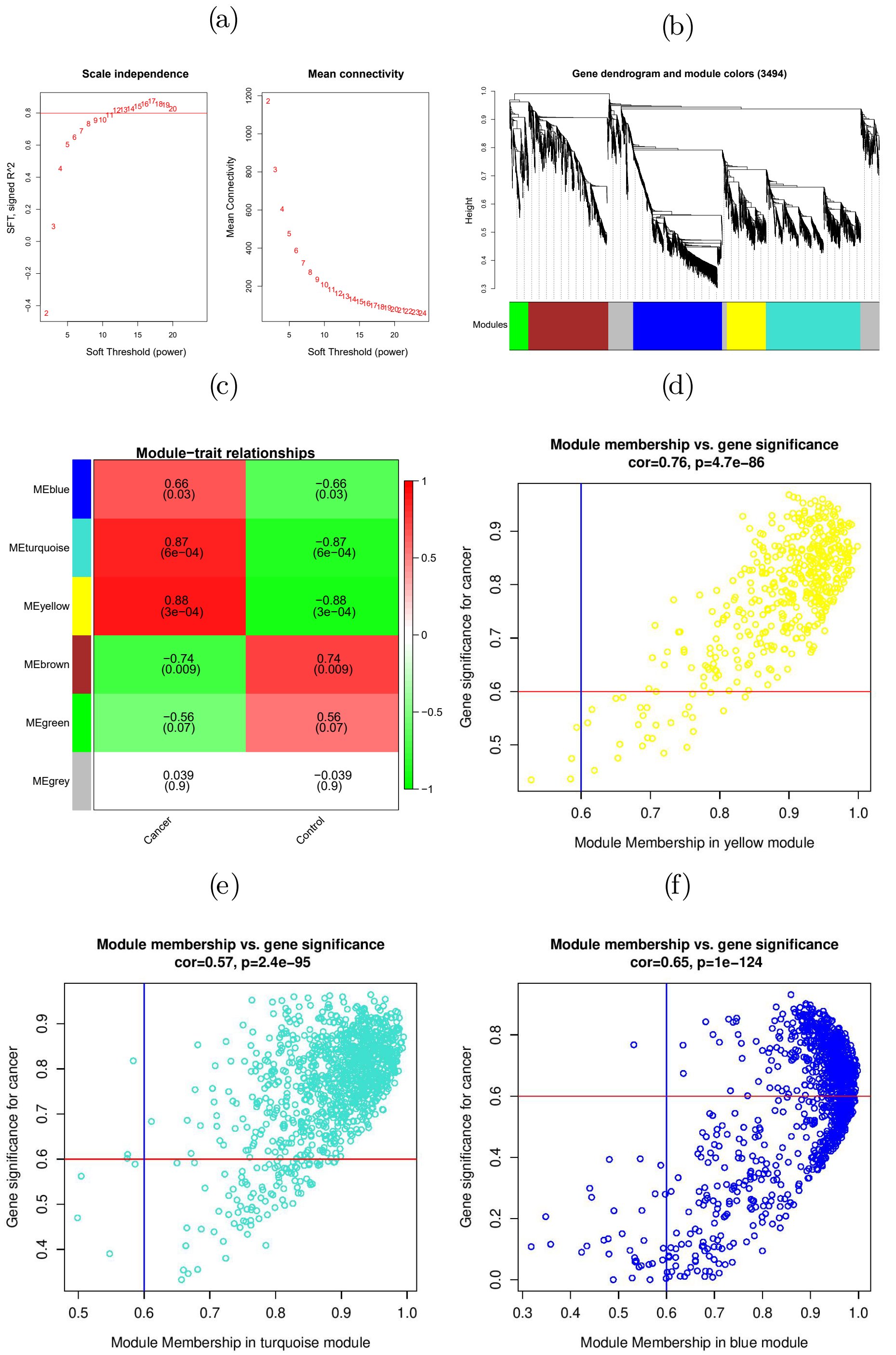

Figure (S14) Plots of Dataset E-MTAB-3494 **(a)** β value from 2 to 20 was evaluated to obtain scale-free network on $R^2 \geq 0.8$. The β of 12 was determined on the basis of scale independence plot. **(b)** Gene tree (dendogram) along with final modules with module colors. Total 6 modules were formed on SFT of 12. **(c)** Modules correlation plot with pancreatic cancer and control, along with correlation values and p-values for all modules. **(d)** Scatter plot for module membership vs. gene significance for PC in the yellow module. **(e)** Scatter plot for module membership vs. gene significance for PC in the turquoise module. **(f)** Scatter plot for module membership vs. gene significance for PC in the blue module.
